# Genome-Wide Scan for Signatures of Human Population Differentiation and Their Relationship with Natural Selection, Functional Pathways and Diseases

**DOI:** 10.1371/journal.pone.0007927

**Published:** 2009-11-20

**Authors:** Roberto Amato, Michele Pinelli, Antonella Monticelli, Davide Marino, Gennaro Miele, Sergio Cocozza

**Affiliations:** 1 Gruppo Interdipartimentale di Bioinformatica e Biologia Computazionale, Università di Napoli “Federico II” - Università di Salerno, Naples, Italy; 2 Dipartimento di Scienze Fisiche, Università degli Studi di Napoli “Federico II”, Naples, Italy; 3 Dipartimento di Biologia e Patologia Cellulare e Molecolare “L. Califano”, Università degli Studi di Napoli “Federico II”, Naples, Italy; 4 Istituto di Endocrinologia ed Oncologia Sperimentale, CNR Napoli, Naples, Italy; 5 Istituto Nazionale di Fisica Nucleare – Sezione di Napoli, Naples, Italy; University of California, Riverside, United States of America

## Abstract

Genetic differences both between individuals and populations are studied for their evolutionary relevance and for their potential medical applications. Most of the genetic differentiation among populations are caused by random drift that should affect all loci across the genome in a similar manner. When a locus shows extraordinary high or low levels of population differentiation, this may be interpreted as evidence for natural selection. The most used measure of population differentiation was devised by Wright and is known as fixation index, or F_ST_. We performed a genome-wide estimation of F_ST_ on about 4 millions of SNPs from HapMap project data. We demonstrated a heterogeneous distribution of F_ST_ values between autosomes and heterochromosomes. When we compared the F_ST_ values obtained in this study with another evolutionary measure obtained by comparative interspecific approach, we found that genes under positive selection appeared to show low levels of population differentiation. We applied a gene set approach, widely used for microarray data analysis, to detect functional pathways under selection. We found that one pathway related to antigen processing and presentation showed low levels of F_ST_, while several pathways related to cell signalling, growth and morphogenesis showed high F_ST_ values. Finally, we detected a signature of selection within genes associated with human complex diseases. These results can help to identify which process occurred during human evolution and adaptation to different environments. They also support the hypothesis that common diseases could have a genetic background shaped by human evolution.

## Introduction

Genetic differences are present in humans at both individual and population level. Human genetic variations are studied for their evolutionary relevance and for their potential medical applications. This studies can help scientists in understanding ancient human population migrations as well as how selective forces act on the human being [1], [2].

According to the theory of neutral variation, most of the genetic variability within species are caused by random drift of selectively neutral polymorphic alleles [3]. Genetic drift should affect all loci across the genome in a similar manner. Therefore, when a locus shows extraordinary high or low levels of variability this may be interpreted as evidence for natural selection [4]. High levels of population differentiation can suggest the acting of a positive selection of advantageous alleles in one or more populations. On the contrary, lower levels of population differentiation can be considered as the effect of balancing selection that tends to maintain a constant proportion of alleles across all populations [5].

Population differentiation is sensitive to a variety of demographic factors (including the rate of drift within populations and the extent of gene flow among them), making it difficult to rule out demographic scenarios that could account for the observed variations. Another class of tests is aimed to detect signature of natural selection by comparing data from different species. These tests explore the fact that mutations can be synonymous and non synonymous, and that non-synonymous mutations are more likely to have an effect on individual fitness. This method is also known as d_N_/d_S_. Results obtained by this comparative approach are rarely interpreted in terms of population genetics theory [6].

The human population is also not homogeneous in terms of disease susceptibility. Risks of common diseases are substantially different among ethnic groups [7]. The understanding of population genetic differentiation, especially in genes associated with diseases, can help to explain the observed variations in the prevalence of diseases. It is not difficult to forecast that, in the future, genetic structure of populations can be used in public health management [8]. Moreover, natural selection on genes that underlie human disease susceptibility has been invoked. In this framework, ancestral alleles reflect ancient adaptation. With the shift in the environment, these alleles increase the risk for common diseases [9].

Different strategies to quantify the population genetic differentiation have been elaborated [10]–[16]. One of the most used is a measure devised by Wright and known as fixation index, or F_ST_ [17], [18], which is the amount of genetic variation among groups relative to a panmictic state. As a test of selection, observed F_ST_ values are compared to those expected under neutrality. The main difficulty of this approach is to determine the distribution of F_ST_ values under neutrality [10]. Recently, however, the abundance of genetic data available allows the creation of an empirical genome-wide distribution to be used for the comparisons. Rather than statistically testing specific loci, we can use their position relative to this distribution to gain insights about their selective histories. In addition, the abundance of information about variability of many genes makes it possible to analyze not only single genes, but also sets of functionally related genes. International HapMap Project [19] by supplying data of a large number of Single Nucleotide Polymorphisms (SNPs) across many human populations, is providing an exceptional tool for studying the genetic structure of human populations.

In the present article we report the results of a genome-wide estimation of F_ST_ on 3,917,301 SNPs from the latest release of HapMap data. Our results show a heterogeneous distribution of F_ST_ values among genomic regions. Furthermore, we studied the relationship between F_ST_ and an evolutionary measure obtained by a comparative interspecific approach. We applied a gene set approach, widely used for microarray data, to detect biochemical pathways under selection. Finally, we detected a signature of selection within genes associated with complex diseases.

## Results

Using F_ST_, we estimated populations differentiation for 3,917,301 SNPs in population samples from the International HapMap Project data (Public release 27, merged II + III). To retain the largest number of SNPs broadly reflecting a continental subdivision, we used data from Yoruba (Africa), Japanese (Asia), Han Chinese (Asia) and CEPH (European descendant) individuals. Combining data from these populations we were able to compare the largest set of genotyped SNPs up to now available. We pooled Japanese and Han Chinese samples due to their geographical closeness. Furthermore, this pooling allowed us to compare our data with previous studies [20], [11]. F_ST_ was estimated according to Weir and Cockerham [18], [21].

**Figure 1:**
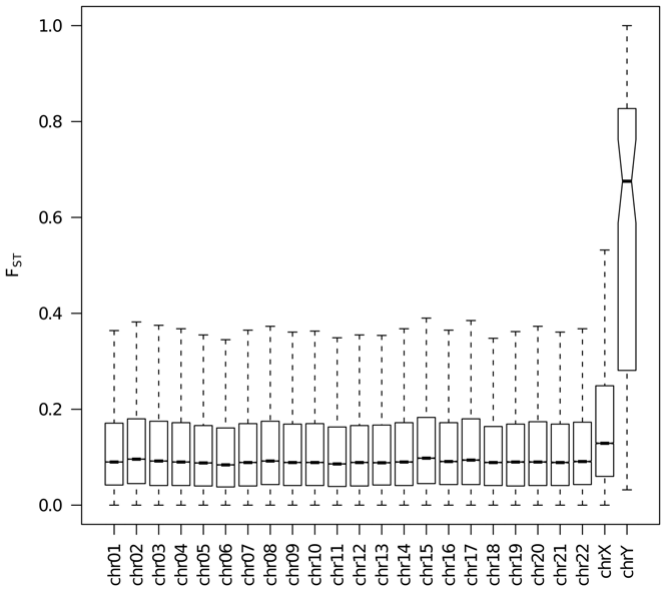
Distribution of F_ST_ values across chromosomes. For each chromosome, the box length is the interquartile range while the horizontal line inside it is the value of the median. The whiskers extend to the most extreme data point <1.5 times the interquartile range from the box. Extremes of the notches represents 95% confidence interval of the median.

After exclusion for Minor Allele Frequency (MAF), we obtained a final SNP sample of 2,125,440 SNPs. The mean F_ST_ was 0.122 (SE  = 5×10^−5^, median  = 0.091, interquartile range  = 0.131; see Supporting Information S1 for more detailed statistics). Figure 1 shows distribution of F_ST_ values for each chromosome. The median F_ST_ values of SNPs on the autosomal and sexual chromosomes were statistically different (Kruskal-Wallis test, p-value <10^−16^). The median F_ST_ values for X and Y chromosomes were 0.129 (mean  = 0.174) and 0.676 (mean  = 0.606) respectively and were notably higher than those of autosomal chromosomes. Also medians between autosomal chromosomes showed significant differences, but in a very small range of values (median range  = 0.084 to 0.098).

**Figure 2:**
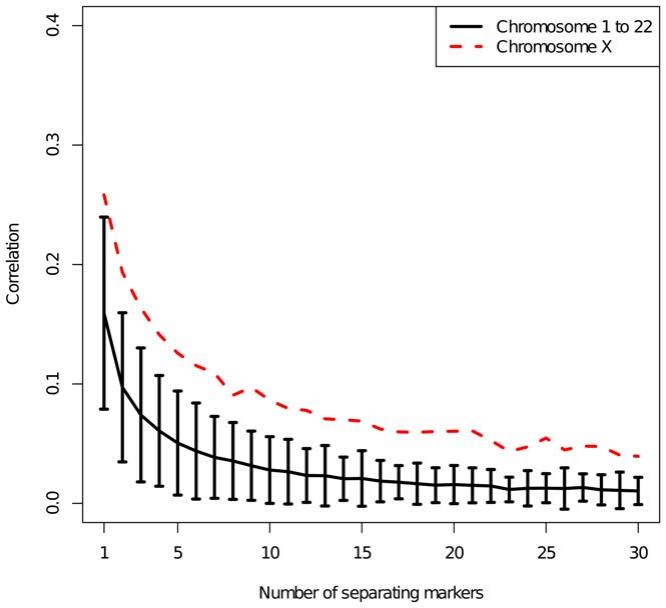
Correlation between F_ST_ values. The correlation is calculated, for each chromosome, for all pairs of SNPs separated by a fixed number of intervening SNPs. Black line shows mean value and 2σ error bars of the correlation of SNPs belonging to autosomal chromosomes. Red line shows correlation among X-linked SNPs.

For each chromosome, we computed the correlations of all pairs of F_ST_ values for neighbouring SNPs separated by a fixed number of SNPs (1 to 30). This method is commonly used to assess whether F_ST_ values are non randomly distributed across chromosomes [4], [22]. As expected, we found that correlation plots are different from those expected from a noisy signal (Figure 2). Moreover, scrambling F_ST_ values across each chromosome produced vanishing correlation values demonstrating that the distribution of data is non-random (data not shown). This result was also supported by a test for non-randomness of data (Ljung-Box test, p-value <10^−16^). Figure 2 shows a clear difference between correlation plots of autosomal and X-linked SNPs, the latter showing higher autocorrelation values. Chromosome Y was excluded from this analysis because of the small number of SNPs sampled.

To attribute F_ST_ value to genes we followed the approach by Akey et al. and Pikrell et al. [4], [16], considering F_ST_ of a gene the maximum F_ST_ value in the gene region (see Material and Methods). It is worth stressing that this approach is very conservative for genes with low F_ST_ values.

**Figure 3:**
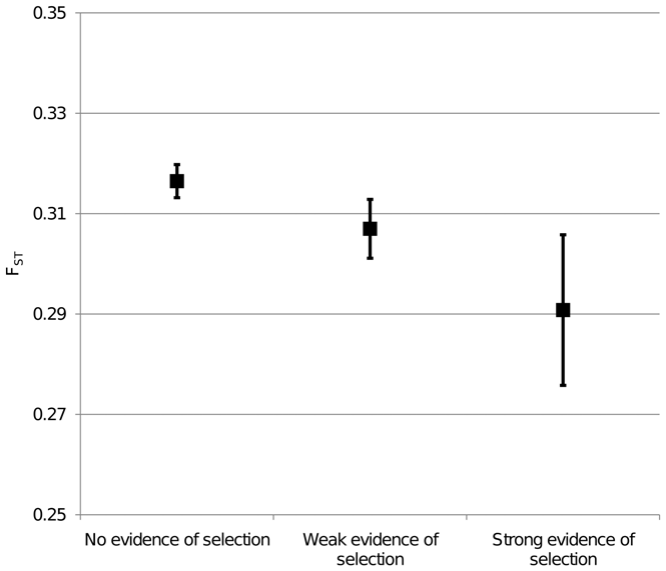
Mean F_ST_ value of genes with and without interspecific evidence of positive selection. Genes were grouped according to the strength of evidence of their positive selection across six species [23]. Vertical bars represent 95% confidence interval.

Selection affects both interspecific (between-species) and intraspecific (within-species) variability. F_ST_ is a measure of intraspecific variability. Estimation of genic d_N_/d_S_ is an interspecific measure of variability [6]. We compared the gene F_ST_ values that we obtained with previously reported data from a genome-wide estimation of genic d_N_/d_S_ [23]. In that article the authors divided genes into subgroups with strong, weak and no evidence of positive selection. We compared F_ST_ values of genes belonging to these subgroups. Genes with both weak and strong evidence of positive selection showed lower F_ST_ values than genes with no evidence of positive selection (ANOVA, p-value <0.001; Bonferroni post-hoc, no evidence vs. weak evidence p-value <0.02, no evidence vs. strong evidence p-value <0.005, weak evidence vs. strong evidence  =  N.S.; Figure 3).

**Figure 4:**
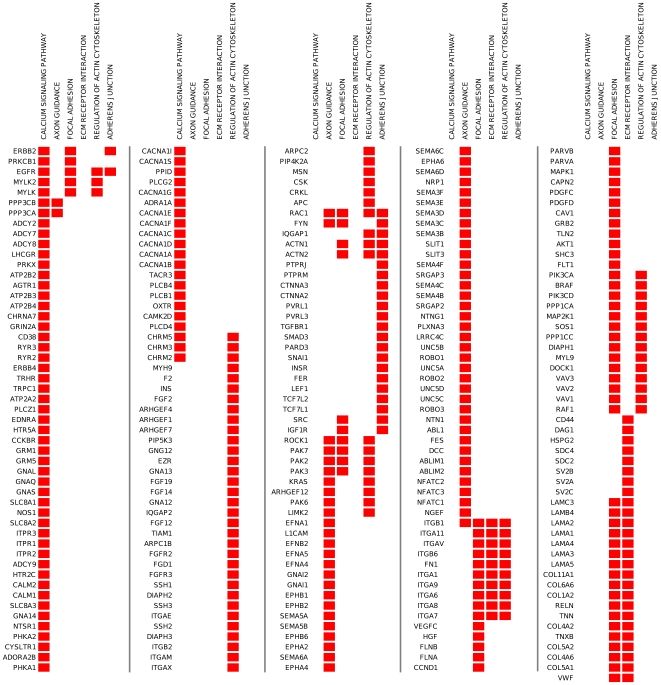
Leading edge genes of the high F_ST_ enriched KEGG pathways identified by GSEA. Genes are indicated by gene symbols. Red box marks the presence of that gene, as leading edge gene, in that pathway.

To identify functions potentially under selective pressure, we used an innovative approach, focusing on gene pathways instead of outliers. We performed this “gene set” analysis using the Gene Set Enrichment Analysis (GSEA) algorithm [24], [25]. GSEA is oriented to identify sets of functionally related genes and is currently used in the analysis of microarray data. Screening the KEGG pathway database by GSEA, we identified 6 KEGG pathways enriched by genes with high values of F_ST_ and one pathway enriched by genes with low values of F_ST_ (Table 1). In this method, the enrichment of a pathway is mainly driven by a group of genes that are called “leading edge genes” (see Material and Methods). Figure 4 shows the leading edge genes for the six pathways with high F_ST_ values. A partial overlap of genes among pathways is present.

**Table 1:** Enriched KEGG pathways identified by GSEA.

Pathway	Name	KEGG ID	Size	FDR
Enriched by high F_ST_ genes				
	Axon guidance	HS04360	126	<0.001
	Focal adhesion	HS04510	194	0.008
	ECM receptor interaction	HS04512	85	0.009
	Regulation of actin cytoskeleton	HS04810	199	0.010
	Adherens junction	HS04520	75	0.010
	Calcium signaling pathway	HS04020	168	0.010
Enriched by low F_ST_ genes				
	Antigen processing and presentation	HS04612	70	0.001

For each pathway is showed the name, the KEGG ID, the number of genes included in the pathway and the p-value after the False Discovery Rate (FDR) correction.

**Figure 5:**
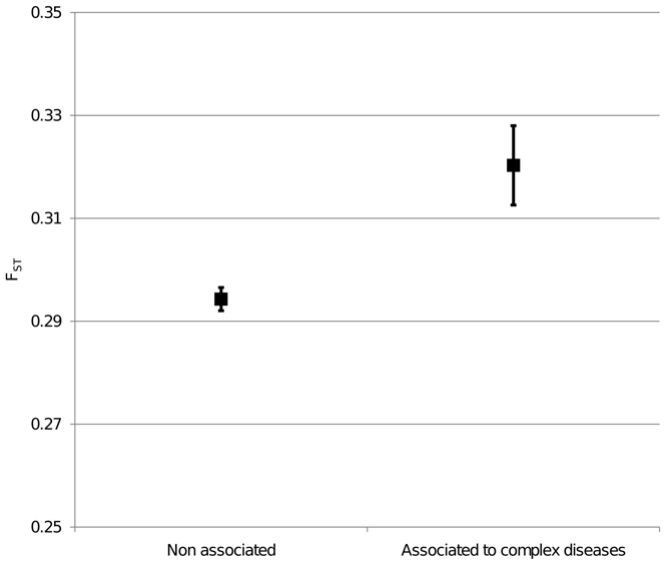
Mean F_ST_ value of genes associated to complex diseases. Genes found positively associated with complex diseases according to the Genetic Association Database are compared with the remaining ones. Vertical bars represent 95% confidence interval.

**Figure 6:**
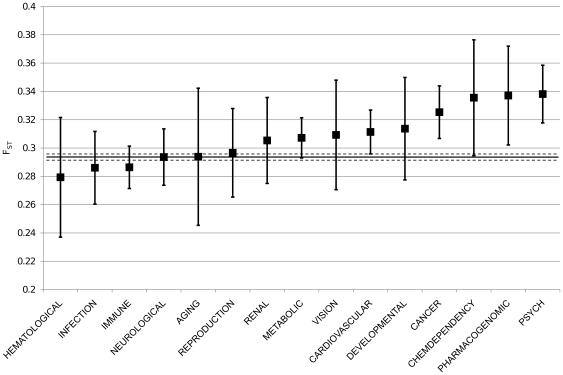
Mean F_ST_ values of genes in different disease classes. Genes were grouped according to the diseases classification of Genetic Association Database. Vertical bars represent 95% confidence interval. Horizontal solid and dashed lines represent mean value and 95% confidence interval of the set of non associated genes.

We then studied populations differentiation of genes associated with complex diseases. We used the Genetic Association Database (GAD) to select genes annotated as having positive association with complex diseases. We compared F_ST_ values of these genes with those where no association had been positively found. Genes associated with complex diseases showed a significant higher mean value of F_ST_ (t-test, p-value <0.001; Moving Block Boostrap, empirical p-value  = 0.0005; Figure 5). Then, we divided diseases in subgroups according to the GAD classification of diseases. Figure 6 shows that large differences of F_ST_ values exist among disease classes, while mean F_ST_ values are usually higher than those of non associated genes.

## Discussion

The study of the evolutionary forces acting in diseases and physiological traits is an exciting field that may drive further researches and, in the future, public health policies. The study of population genetic differentiation could help the understanding of human evolution, demographic history and disease susceptibility [26]. To study population differentiation we performed a genome-wide F_ST_ calculation using the latest available data release from the HapMap. Using this release we were able to increase both the number of SNPs and the number of individuals analysed in comparison to recent analogous studies [15]. We focused on samples from three different continents (Africa, Asia, Europe) to obtain a broad but sound measure of populations differentiation.

We found an overall mean F_ST_ value (0.122) broadly consistent with previous estimations [4], [22], [15]. The slightly higher value that we obtained could be explained by the exclusion of SNPs with MAF <0.05 and the inclusion of heterochromosomes in the calculation. Indeed, as expected [4], we observed a significantly higher median F_ST_ value of X-linked SNPs with respect to the autosomal ones. Furthermore, we found median F_ST_ value of Y-linked SNPs to be significantly higher than both the autosomal and the X-linked ones. Previous data from smaller datasets suggested a similar phenomenon [27], but, in our knowledge, this is the first observation made on Y chromosome F_ST_ in a more robust framework. The higher population differentiation for X and Y chromosomes can be due to various causes: their smaller effective population size (three-quarter and one-quarter of autosomes, respectively), the lower mutation and recombination rates and the different selective pressure between genders have been invoked [4], [6], [28].

Keinan et al. showed that there was a period of accelerated genetic drift on chromosome X associated with the human dispersal out of Africa. In particular, they estimated the autosome-to-X genetic drift ratio between North Europeans and East Asians is consistent with the expected 3/4 while it is significantly reduced between North Europeans and West Africans, and between East Asians and West Africans [29]. As possible explanations they suggested that a gender-biased process reduced the female effective population size, or that an episode of natural selection affecting chromosome X was associated with the founding of non-African populations. Our results are consistent with these finding. We computed population pair-wise F_ST_ and we found that the autosome-to-X genetic drift ratios (Q), estimated as in [29], are compatible with those reported in [29] (Asia-Europe Q = 0.72; Asia-Africa Q = 0.66; Europe-Africa Q = 0.65).

The weak but significant correlation that we found among F_ST_ values of neighbouring markers demonstrated that they are non-randomly distributed along chromosomes. This result confirms previous observations made on smaller datasets [4], [22]. We extended for the first time this observation to the X chromosome and we found that correlation was slightly stronger than that of autosomes. It has been observed that correlation between SNPs is proportional to Linkage Disequilibrium (LD) [22]. Therefore, the higher value of autocorrelation that we found can be explained by the higher value of LD in X chromosomes [22].

Population genetics approach has been largely used for studying natural selection. Other approaches include the comparative one, in which data from different species are used. The most commonly used method is to compare the ratio of nonsynonymous mutations per nonsynonymous site to the number of synonymous mutations per nonsynonymous site (d_N_/d_S_). Data from comparative studies and from population genetics are poorly connected. We found that genes with a high d_N_/d_S_ ratio, indicating positive selection, showed a significantly lower F_ST_ mean value. In our knowledge this represents the first attempt to connect human population genetic data and comparative data at a genome-wide level. Our finding does not conflict with previous studies performed on a restricted number of genes [30]. It is well established that comparative data provides the most unambiguous evidence for selection, but relatively vague assertion on the type of selection and if the selection is currently acting in a population [6]. For such reasons the connection with population genetic data is needed. Further studies, mainly focused on this topic, are required to confirm and understand the relationship that we found.

We used a gene set approach to identify pathways with extraordinary levels of population genetic differentiation. The traditional approach used to perform this analysis is based on the identification of those loci outliers in a given statistic. This approach has been recently reviewed and its limits explored [10], [31]–[33]. Interestingly, similar criticisms are arising on analogous methods used in transcriptomic data analysis. In this field, alternative approaches, as the “gene set” ones, are gaining increasing interest. Among the tools implementing this approach, Gene Set Enrichment Analysis [34], [25] is one of the most used [35], [36]. The key idea underlying GSEA is to focus on gene sets, which are defined as groups of genes sharing common features (e.g. biological pathways, chromosomal position, etc.). In microarray data analysis, GSEA aims to determine whether a gene set shows statistically significant, concordant differences between two biological states or phenotypes. This method has been tailored for microarray data, however its use is being explored also in different fields [37], [38]. To the best of our knowledge, the present report is the first attempt to functionally analyse genes under selective pressure by a gene set statistical approach.

Using very conservative statistics, the GSEA analysis found differential F_ST_ values on seven KEGG pathways, one enriched by low F_ST_ genes and six enriched by high F_ST_ genes. However, it is important to note that the discrepancy between the number of low and high F_ST_ pathways is a consequence of the way by which we attributed F_ST_ values to genes rather than underlying evolutionary forces. The only pathway with decreased degree of differentiation among populations was the “antigen processing and presentation” pathway. Included in this pathway are genes involved in the antigen-presenting machinery as (i) the expression of major histocompatibility complex (MHC) molecules, (ii) the mechanism of cross-presentation, and (iii) the interaction of antigen-presenting cells. Opposing views exist concerning the evolutionary forces that shaped the innate immune system. In particular, the relative impact of purifying and balancing selection is under discussion [39], [40]. Barreiro et al demonstrated that several SNPs of genes related to the immune response to pathogens showed very high F_ST_ values [15]. On the other hand, Akey et al. reported a four times increase of proteins that perform a defense/immunity function in the group of the low F_ST_ genes [4]. Moreover, low levels of population differentiation have been previously detected at loci that are involved with host–pathogen responses (HLA class I and class II genes, beta-globin, *G6PD*, glycophorin A, interleukin 4 receptor-alpha and *CCR5*) [5]. Further evidence arises from the group of genes that we studied and that were previously described to be under positive selection. This group of genes, which we found with low F_ST_ values, was described to be enriched for several functions related to immunity and defence [23].

Among the six gene sets enriched by high F_ST_ genes, we found the “calcium signalling” pathway.

Calcium is the most abundant mineral in the body. It is also a highly versatile intracellular signal that regulates many cellular processes in response to different external stimuli, as growth factors [41]. We found very high F_ST_ values in three genes belonging to the growth factor stimulated calcium signalling pathway, namely *EGFR*, *ERBB2*, and *ERBB4*. It is interesting to note that a previous study from Pickrell et al. found that *ERBB4* showed extreme signs of haplotype selective sweep in non-African populations [16]. The authors suggested that this gene could affect an unidentified phenotype that experienced a strong recent selection in non-African population. Our gene set approach seems to confirm this finding and expands this observation to other members of the *ERBB* gene family.

The other five high F_ST_ pathways are involved in the control of cell shape and mobility. Among them, four interconnected pathways (“focal adhesion”, “regulation of the actin cytoskeleton”, “adherens junction” and “extra cellular matrix receptor interaction”) govern growth-related processes and morphogenesis. Morphological traits have been demonstrated to show strong signature of positive selection [15]. These pathways were found also to be altered in a mouse model of fetal alcohol syndrome, associated with a low birth-weight phenotype [42]. Indeed, human body shape and size varies among populations showing a correlation with geographic and climate variables [43]. In addition, in the “adherens junction” pathway, one of the strongest F_ST_ values was showed by *TCF7L2*, the gene with largest type 2 diabetes effect size found to date [44]. This last finding is consistent with previous observations [44], [16]. Since it has been demonstrated that *TCF7L2* variants also substantially influence normal birth-weight variations [45], a complex interplay between pathways that govern growth-related processes and susceptibility to type 2 diabetes could be hypothesized.

The last high F_ST_ pathway, the “axon guidance”, is involved in brain wiring during foetal development and repair throughout life. Axon guidance proteins and their relative binding partners have also an emerging role in the pathogenesis of several neurodegenerative and psychiatric diseases such as schizophrenia [46], [47]. Signature of recent positive selection inferred by identification of selective sweeps in specific populations was found in genes involved in schizophrenia [48]. Moreover, population dependent results were obtained when gene-association studies were performed using several high F_ST_ genes present in this gene set [49], [50].

It has been suggested that alleles involved in common disease could be targets of selection [51], [9], [52], [43]. The common disease/common variant (CD/CV) hypothesis proposes that common diseases are usually caused by one or a few common disease susceptibility alleles. These genetic variants represent ancestral alleles, presumably under selective pressure, that have become disadvantageous after changes in environment and of lifestyle [51], [53], [54]. We found that genes associated with complex diseases showed a significant higher mean value of F_ST_, supporting the CD/CV hypothesis. However, several previous studies of SNPs associated with complex diseases did not find significant evidence of population differentiation [55], [56]. On the other hand, further studies observed that the distribution of maximum F_ST_ was shifted upward in regions associated with type 2 diabetes mellitus [16]. Moreover SNPs known to protect against obesity and diabetes showed very high F_ST_ values [15]. Simulation studies also provided support for the CD/CV hypothesis [57].

According to the GAD classification of diseases, we divided the overall group of the genes associated with complex diseases. Clear differences in F_ST_ means among the various classes were present. In particular, several disease classes, namely “hematological”, “infection”, and “immune”, showed an F_ST_ mean value slightly lower than the mean value of non-associated genes. Nevertheless, the majority of the classes showed F_ST_ mean values to be higher than the non-associated one. Highest F_ST_ values were detected in “pharmacogenomics” and “psychiatric” classes. GAD classifies in “pharmacogenomics” those diseases related to drug effects. It is well established that drugs effects are race/ethnic specific [58]. The GAD “psychiatric” class includes mental disorders. Why genes that confer susceptibility to mental diseases are still maintained by natural selection, is an old question which, up to now, is still unanswered. The compensatory advantage for genes associated to intermediate phenotypes has been invoked as explanation for this phenomenon, also called “psychiatric paradox” [59]. Further studies should be performed to determine if the high level of population differentiation that we found for this disease class could be related to the psychiatric paradox.

The results presented in this paper could contribute to further explorations of the ongoing selection in humans. Further studies are needed to clarify the biological pathways involved and to better elucidate the role of natural selection in human complex diseases.

## Materials and Methods

### Data

All analysis are based on the HapMap Public Release #27 (merged II+III) datafiles (http://www.hapmap.org). We analyzed the data from the CEPH (Utah residents with ancestry from northern and western Europe; CEU, n = 165), Yoruba in Ibadan, Nigeria (YRI, n = 167), Han Chinese in Beijing, China (CHB, n = 84) and Japanese in Tokyo, Japan (JPT, n = 86) samples. We pooled the CHB and JPT samples to form a single sample. Additional SNP information about physical positions and SNP-gene association were obtained from dbSNP build 129 (http://www.ncbi.nlm.nih.gov/projects/SNP). In particular, according to dbSNP classification, we considered all SNPs within 2 kb of a gene (locus region) as associated to that gene. Data from the International HapMap Project and dbSNP were merged in a local MySQL database by a set of script from Amigo et al. [60]. When we consider the whole Hap map dataset (autosomes and heterochromosomes) we analyzed a total of 3,917,301 SNPs.

We excluded by this analysis SNPs that were non sampled or non polymorphic in all the three samples. We excluded also SNPs with a minor allele frequency <5% in any of the 3 samples, getting a final SNP sample of 2,125,440 SNPs.

### Estimates of F_ST_

Fixation index (F_ST_) was calculated using the unbiased estimator proposed by Weir and Cockerham [18], [21]. We implemented this calculation in a Perl script available upon request by the authors.

All analyses presented in this work were also performed by using the original F_ST_ estimator proposed by Wright [17] and results are almost identical to that obtained by the Weir and Cockerham method. This result is not surprising considering previous reports [61], [4] and the strong correlation that we found between these two measures (Spearman's ρ = 0.97, p<10^−16^; see Supporting Information S1).

The maximum F_ST_ values among those of the SNPs associated to the gene according to dbSNP (see above) was used to assign a F_ST_ value to each gene. This approach is consistent with previously described ones [4], [16]. We studied the correlation between F_ST_ value and gene length and we found that the former have a quite marginal effect on the latter (R^2^ = 0.2).

### Statistical Analysis

SNPs F_ST_ values are not normally distributed across chromosomes. Thus to detect differences among medians F_ST_ values of chromosomes we used the non-parametric Kruskal-Wallis test. Conversely, F_ST_ values of genes are normally distributed (Kolmogorov-Smirnov/Lilliefor test, p<0.001,) thus comparison among these values were performed by using parametric tests (ANOVA and t-test).

All statistical analyses were performed with R ver. 2.9 (R Foundation for Statistical Computing, Vienna, Austria; http://www.r-project.org/). Non-randomness of data was assessed by using a Ljung-Box test (R function “Box.test”). We calculated the autocorrelation of each chromosome which can be seen as the mean correlation of all pairs of F_ST_ values separated by a fixed number of values (R function “acf”).

A list of about 4000 genes positively selected was obtained from the track “Positively Selected Genes” (database “hg18”, table “mammalPsg”) in UCSD Genome browser (http://genome.ucsc.edu). This list was produced by a genome wide scan in six mammalian genomes performed by Kosiol et al. [23]. In particular they identified (i) 400 genes with strong evidence of positive selection across species, (ii) 144 genes with strong evidence of positive selection in one or more branches, (iii) 3705 genes with weak evidence of positive selection on one or more branches, and (iv) 12280 (orthologs) genes with no significant evidence of positive selection. We pooled first and second group into a single “strong evidence of positive selection” group. Differences among groups were evaluated by ANOVA with Bonferroni post-hoc calculation.

Genes associated with complex diseases were obtained from the Genetic Association Database (GAD; October 1 2007 update; http://geneticassociationdb.nih.gov). We only kept genes with positive evidence of association, for a total of 1789 genes. According to GAD, these genes are divided into 15 classes of diseases. We excluded from the analysis four diseases classes (Other, Unknown, Mitochondrial and Normal variations) because they were not informative. Differences among groups were evaluated by a t-test and a resampling approach. In particular, we used a Moving Block Boostrap (MBB) strategy [62]. Briefly, (i) we resampled 10000 times 1789 set of adjacent SNPs {n_i_}_j_ with i = 1, …,1789 and j = 1, …,10000 and with each set n_i_ having the same number of SNPs as the i-th GAD associated gene; (ii) for each resample, we computed the F_ST_ of each set n_i_ according to our method (the maximum F_ST_ values among those of the SNPs in the set); then, (iii) we computed the mean F_ST_ value of each resample j obtaining a distribution to which compare the mean F_ST_ value of the GAD associated genes.

### Functional Analysis

We used Gene Set Enrichment Analysis (GSEA) 2.0 [63] to detect KEGG pathways enriched by genes with low or high values of F_ST_. We provided GSEA, by its “Preranked” feature, with a list L of genes ranked according to their F_ST_ value. Given an a priori defined set of genes S representing a pathway (e.g., genes encoding products in a metabolic pathway), the goal of GSEA is to find out whether the members of S are randomly distributed throughout L or mainly found at the top or bottom (i.e. being “enriched”). Since GSEA preferably expect the values to rank for (in our case F_ST_) to vary from negative to positive values, we linear shifted these values to get vanishing mean.

We explored the enrichment of KEGG pathways included in the software. For each pathway a False Discovery Rate (FDR) is computed, representing the statistical significance of the enrichment. For experimental conditions similar to the ours, GSEA user's guide suggests a threshold of significance FDR ≤0.05. Because of the exploratory nature of this study, we used a more conservative threshold of significance (FDR ≤0.01). Overlap among pathways was examined by the “Leading edge analysis” feature of GSEA.

## Supporting Information

Supporting Information S1 Additional figures and tables
(1.07 MB PDF)
