# Supplementary material for: Genome-Wide Scan for Signatures of Human Population Differentiation and Their Relationship with Natural Selection, Functional Pathways and Diseases
Source: PLoS One. 2009 Nov 20;4(11):e7927. doi: 10.1371/journal.pone.0007927 (PMC2775949; doi:10.1371/journal.pone.0007927)
Supplement: Supporting Information S1 — Additional figures and tables (1.07 MB PDF) [file pone.0007927.s001.pdf]

Genome wide scan for signatures of human population differentiation and their relationship with natural selection, functional pathways and diseases

Roberto Amato[1], Michele Pinelli, Antonella Monticelli, Davide Marino, Gennaro Miele and Sergio Cocozza

Supplementary material

Wright vs. Weir&Cockerham F_{ST} 2
Per-chromosome statistics of F_{ST} values 3
Enriched pathways 4
Leading edge 11
GAD diseases classification 18

[1] *roamato@na.infn.it*

Weir&Cockerham vs. Wright F_{ST}

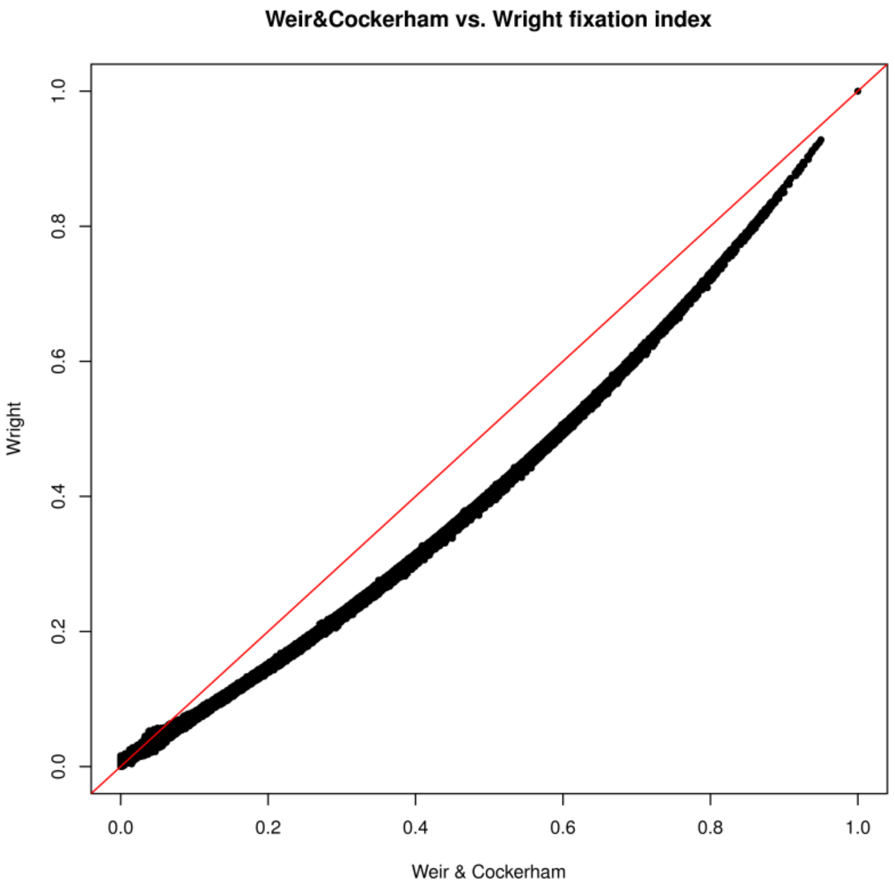

For each SNP, we computed its F_{ST} value using both the Wright and the Weir&Cockerham estimators. As expected, the two measures are strongly correlated (Spearman's ρ=0.97, $p<10^{-16}$).

Per-chromosome statistics of F_{ST} values

Chromosome	Number of SNPs	Mean	Standard Deviation	Median	Interquartile range
1	160631	0.121	0.110	0.090	0.129
2	182756	0.127	0.114	0.096	0.135
3	141273	0.123	0.111	0.092	0.134
4	131470	0.122	0.112	0.090	0.131
5	138353	0.118	0.107	0.088	0.126
6	142779	0.114	0.104	0.084	0.123
7	114767	0.120	0.110	0.089	0.130
8	119383	0.123	0.110	0.092	0.132
9	97952	0.120	0.108	0.089	0.128
10	110485	0.120	0.110	0.089	0.129
11	106880	0.115	0.105	0.086	0.124
12	96493	0.119	0.108	0.089	0.126
13	83084	0.117	0.104	0.089	0.125
14	67477	0.120	0.107	0.090	0.131
15	59040	0.130	0.116	0.098	0.138
16	59604	0.121	0.108	0.091	0.129
17	50163	0.127	0.114	0.094	0.137
18	62453	0.116	0.103	0.089	0.123
19	32325	0.119	0.109	0.090	0.129
20	51481	0.123	0.113	0.090	0.133
21	28532	0.120	0.110	0.089	0.128
22	26757	0.122	0.109	0.091	0.130
X	61204	0.174	0.153	0.129	0.189
Y	98	0.606	0.282	0.676	0.528
Overall	*2125440*	*0.122*	*0.111*	*0.091*	*0.131*

Enriched pathways

KEGG pathways found enriched by using GSEA. Genes are coloured according to their FST value through the "Color objects in KEGG pathways" functionality provided by KEGG (http://www.genome.jp/kegg/tool/color_pathway.html).

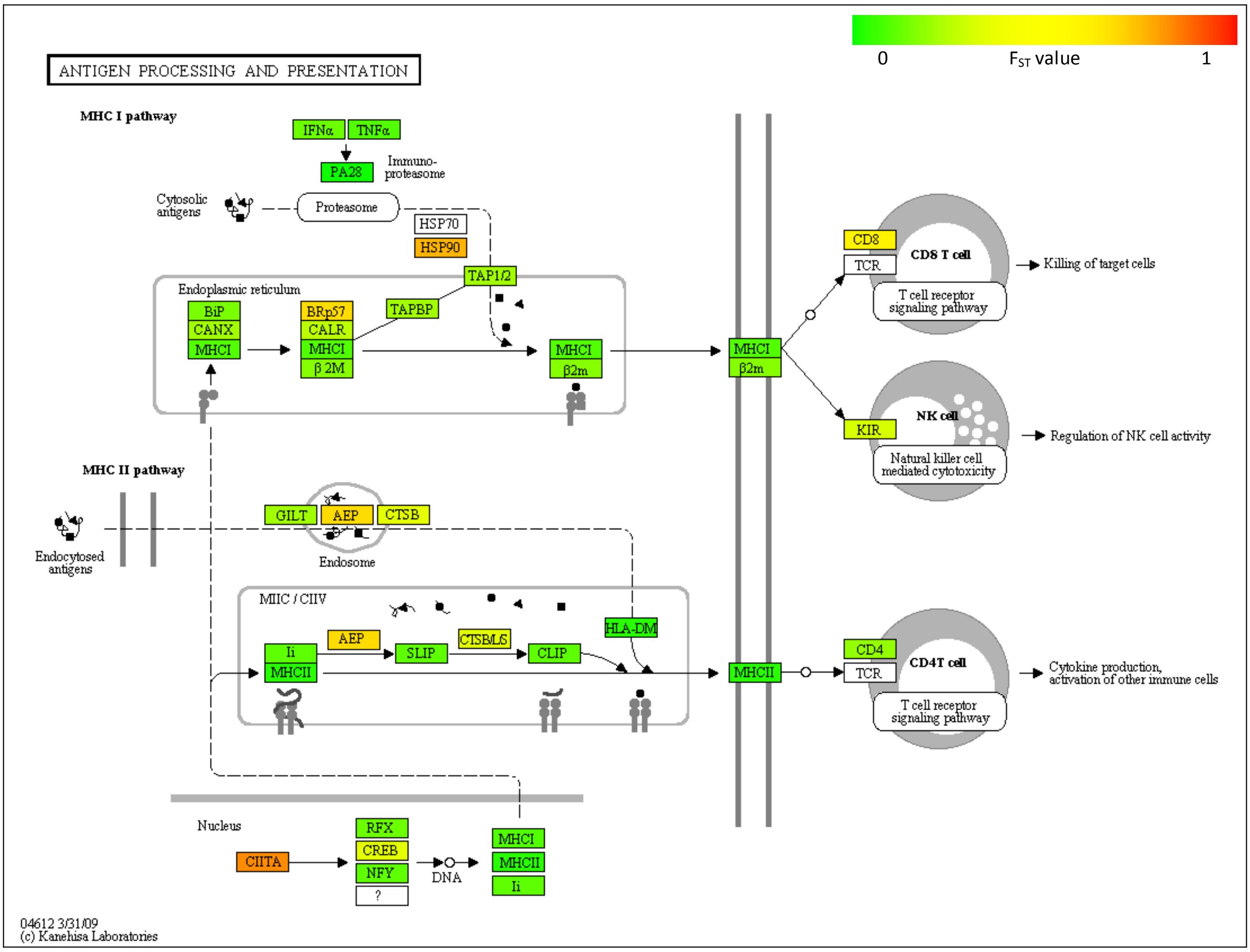

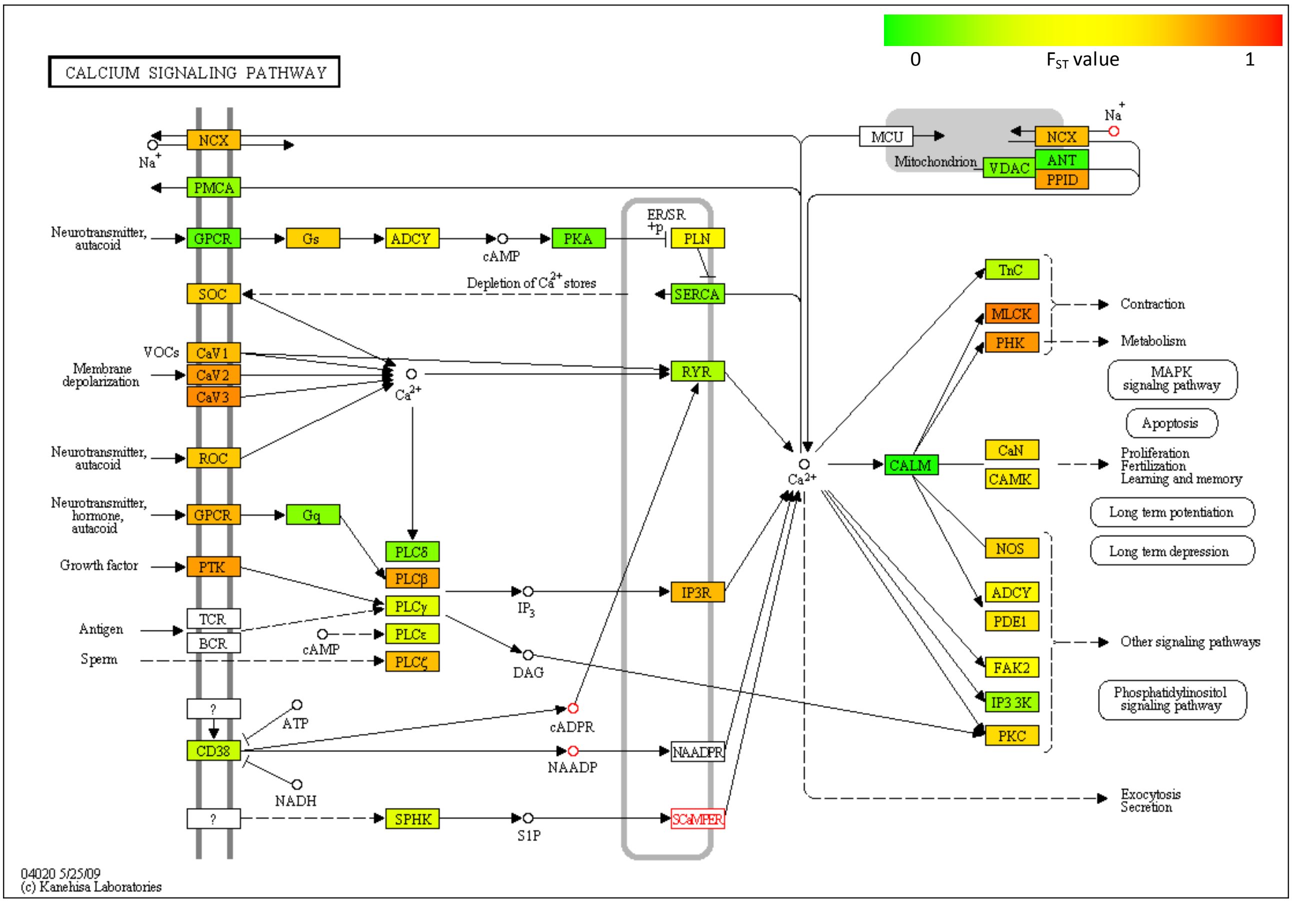
CALCIUM SIGNALING PATHWAY
0
FST value
1
NCX
Na+
PMCA
Neurotransmitter, autacoid
GPCR
Gs
ADCY
cAMP
PKA
ER/SR
+p
PLN
SOC
Depletion of Ca2+ stores
SERCA
VOCs
CaV1
CaV2
CaV3
Membrane depolarization
Ca2+
RYR
ROC
Neurotransmitter, hormone, autacoid
Gq
PLCδ
PLCβ
PLCγ
PLCε
PLCζ
Growth factor
PTK
Antigen
TCR
BCR
Sperm
IP3
IP3R
DAG
ATP
cADPR
CD38
NAADP
NAADPR
NADH
SPHK
S1P
SCaMPER
MCU
Mitochondrion
VDAC
ANT
PPID
TnC
MLCK
PHK
Contraction
Metabolism
MAPK signalng pathway
Apoptosis
CALM
CaN
CAMK
Proliferation
Fertilization
Learning and memory
Long term potentiation
Long term depression
NOS
PDE1
FAK2
IP3 3K
PKC
Other signaling pathways
Phosphatidylinositol signaling pathway
Exocytosis
Secretion
04020 5/25/09
(c) Kanehisa Laboratories

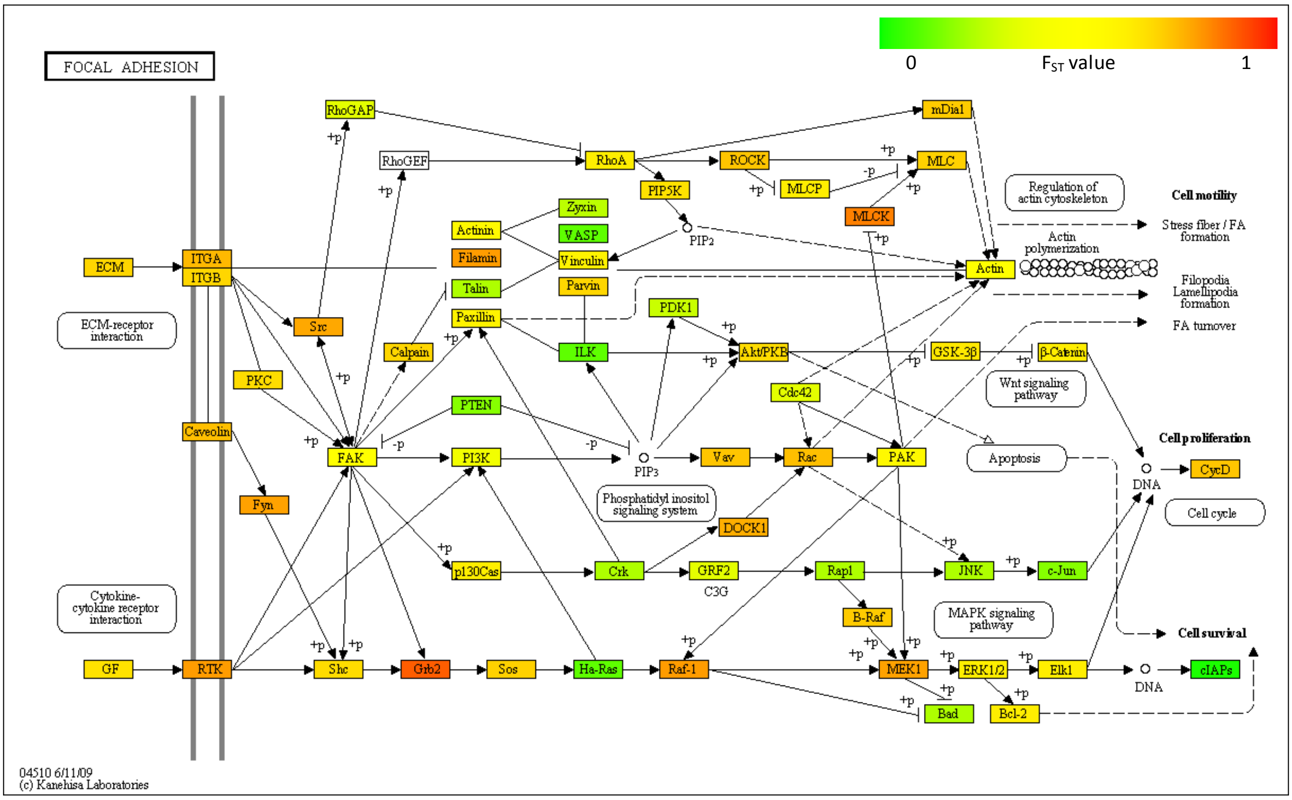
FOCAL ADHESION
0
FST value
1
ECM
ITGA
ITGB
ECM-receptor interaction
Cytokine-cytokine receptor interaction
GF
RTK
Caveolin
PKC
Fyn
Src
RhoGAP
RhoGEF
RhoA
ROCK
MLCP
MLC
MLCK
mDia1
PIP5K
PIP2
Actinin
Filamin
Talin
Zyxin
VASP
Vinculin
Parvin
Paxillin
Calpain
ILK
PDK1
Akt/PKB
GSK-3β
β-Catenin
Wnt signaling pathway
PTEN
FAK
PI3K
PIP3
Phosphatidyl inositol signaling system
Vav
Rac
PAK
Cdc42
DOCK1
p130Cas
Crk
GRF2
C3G
Rap1
B-Raf
JNK
c-Jun
MAPK signaling pathway
Shc
Grb2
Sos
Ha-Ras
Raf-1
MEK1
ERK1/2
Elk1
Bad
Bcl-2
Actin
Actin polymerization
Regulation of actin cytoskeleton
Cell motility
Stress fiber / FA formation
Filopodia Lamellipodia formation
FA turnover
Apoptosis
Cell proliferation
DNA
CycD
Cell cycle
Cell survival
cIAPs
04510 6/11/09
(c) Kanehisa Laboratories

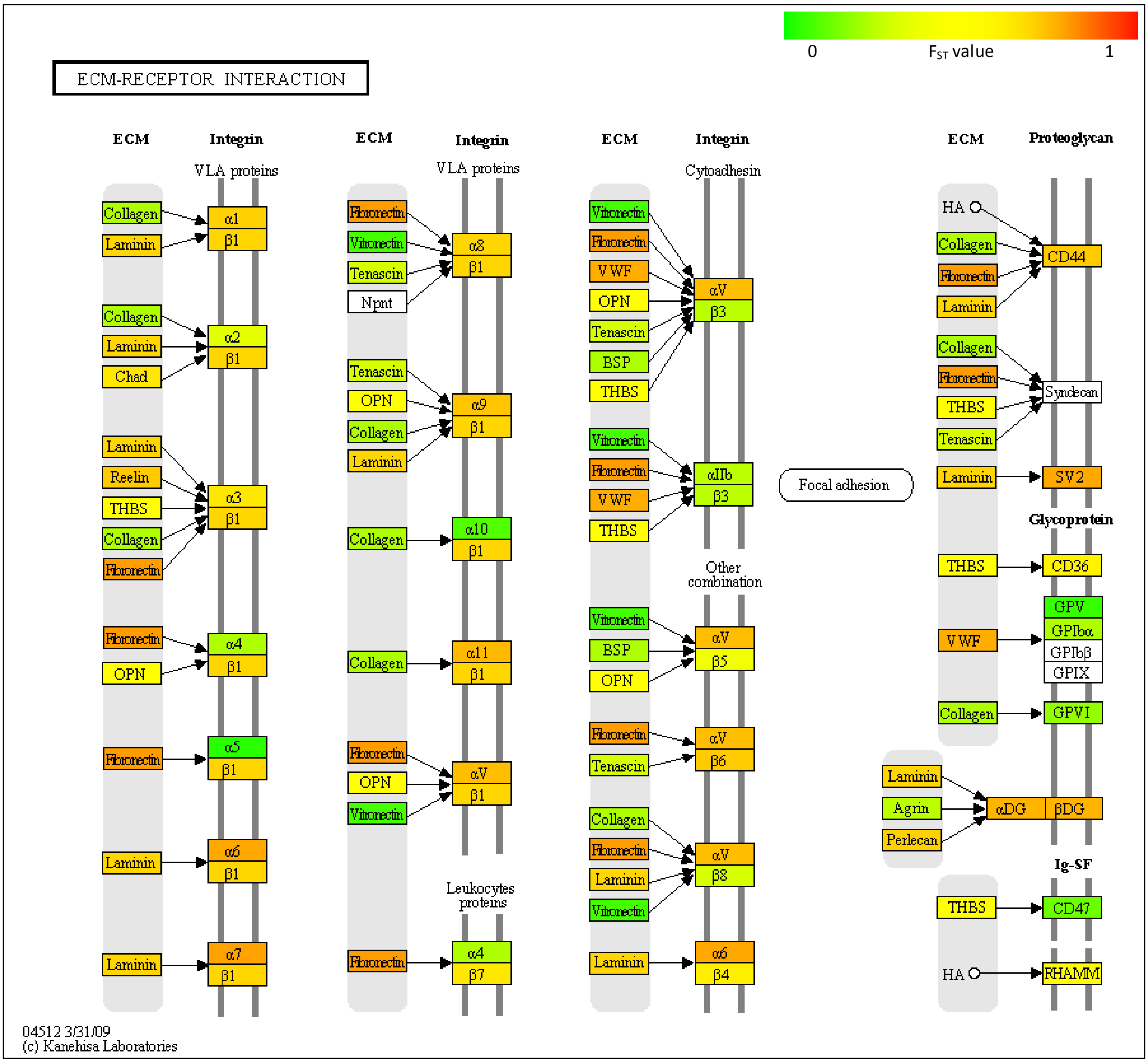
0
FST value
1
ECM-RECEPTOR INTERACTION
ECM
Integrin
VLA proteins
Collagen
Laminin
α1
β1
Collagen
Laminin
Chad
α2
β1
Laminin
Reelin
THBS
Collagen
Fibronectin
α3
β1
Fibronectin
OPN
α4
β1
Fibronectin
α5
β1
Laminin
α6
β1
Laminin
α7
β1
ECM
Integrin
VLA proteins
Fibronectin
Vitronectin
Tenascin
Npnt
α8
β1
Tenascin
OPN
Collagen
Laminin
α9
β1
Collagen
α10
β1
Collagen
α11
β1
Fibronectin
OPN
Vitronectin
αV
β1
Leukocytes proteins
Fibronectin
α4
β7
ECM
Integrin
Cytoadhesin
Vitronectin
Fibronectin
VWF
OPN
Tenascin
BSP
THBS
αV
β3
Vitronectin
Fibronectin
VWF
THBS
αIIb
β3
Other combination
Vitronectin
BSP
OPN
αV
β5
Fibronectin
Tenascin
αV
β6
Collagen
Fibronectin
Laminin
Vitronectin
αV
β8
Laminin
α6
β4
Focal adhesion
ECM
Proteoglycan
HA
Collagen
Fibronectin
Laminin
CD44
Collagen
Fibronectin
THBS
Tenascin
Syndecan
Laminin
SV2
Glycoprotein
THBS
CD36
VWF
GPV
GPIbα
GPIbβ
GPIX
Collagen
GPVI
Laminin
Agrin
Perlecan
αDG
βDG
Ig-SF
THBS
CD47
HA
RHAMM
04512 3/31/09
(c) Kanehisa Laboratories

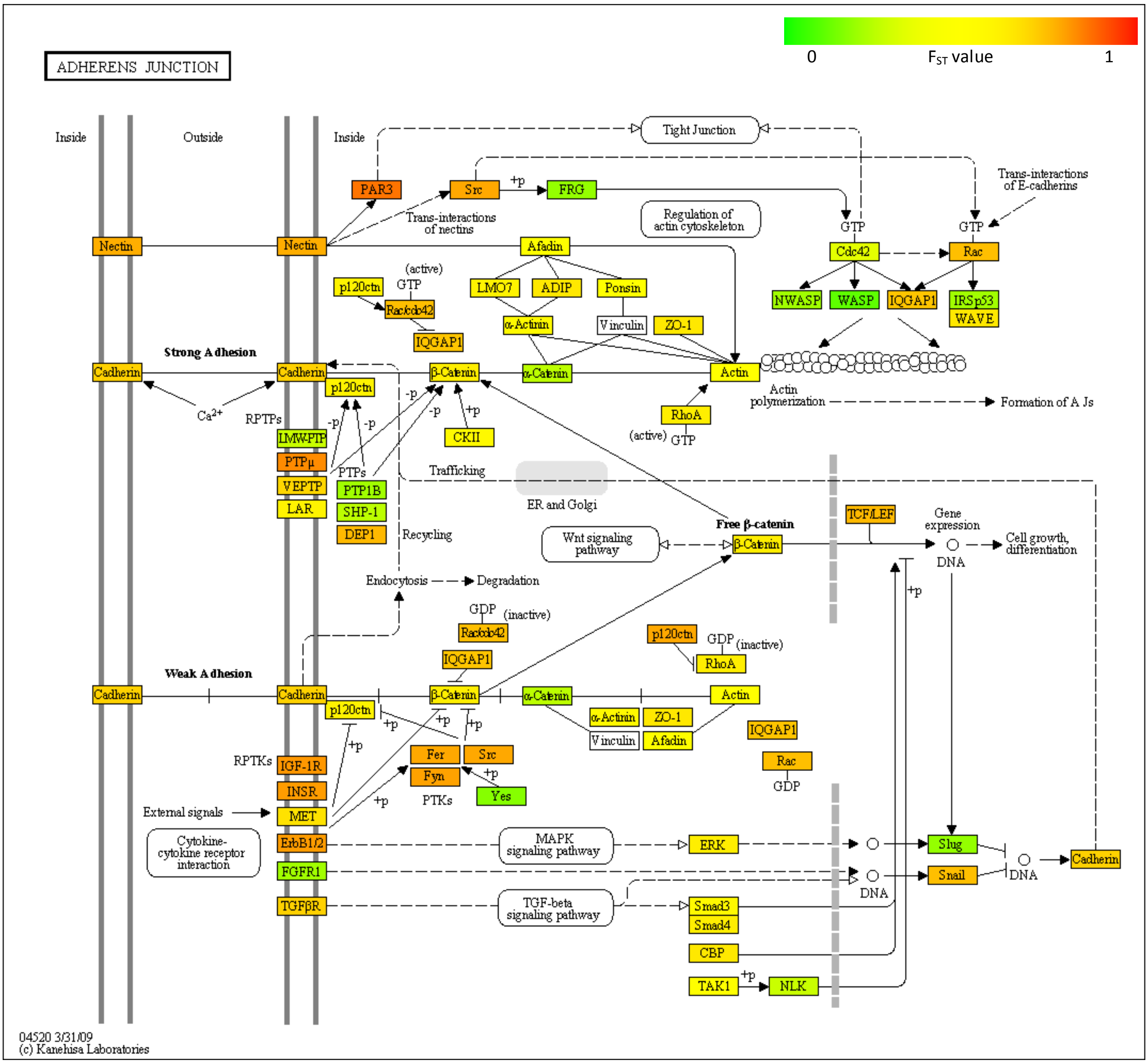

ADHERENS JUNCTION
0
FST value
1
Inside
Outside
Inside
Tight Junction
PAR3
Src
+p
FRG
Trans-interactions of nectins
Regulation of actin cytoskeleton
Trans-interactions of E-cadherins
GTP
GTP
Nectin
Nectin
Afadin
Cdc42
Rac
(active)
GTP
p120ctn
Rac/cdc42
LMO7
ADIP
Ponsin
NWASP
WASP
IQGAP1
IRSp53
WAVE
α-Actinin
Vinculin
ZO-1
IQGAP1
Strong Adhesion
Cadherin
Cadherin
β-Catenin
α-Catenin
Actin
p120ctn
Ca2+
RPTPs
-p
-p
-p
-p
+p
Actin polymerization
Formation of AJs
RhoA
CKII
(active) GTP
LMW-PTP
PTPμ
VEPTP
LAR
PTPs
PTP1B
SHP-1
DEP1
Trafficking
ER and Golgi
Recycling
Free β-catenin
TCF/LEF
Gene expression
Wnt signaling pathway
β-Catenin
Cell growth, differentiation
DNA
Endocytosis
Degradation
+p
GDP
(inactive)
Rac/cdc42
p120ctn
GDP (inactive)
RhoA
IQGAP1
Weak Adhesion
Cadherin
Cadherin
β-Catenin
α-Catenin
Actin
p120ctn
+p
+p
+p
α-Actinin
ZO-1
Vinculin
Afadin
IQGAP1
RPTKs
IGF-1R
Fer
Src
Rac
GDP
Fyn
+p
INSR
Yes
+p
PTKs
External signals
MET
Cytokine-cytokine receptor interaction
ErbB1/2
MAPK signaling pathway
ERK
Slug
FGFR1
Snail
DNA
Cadherin
DNA
TGFβR
TGF-beta signaling pathway
Smad3
Smad4
CBP
TAK1
+p
NLK
04520 3/31/09
(c) Kanehisa Laboratories

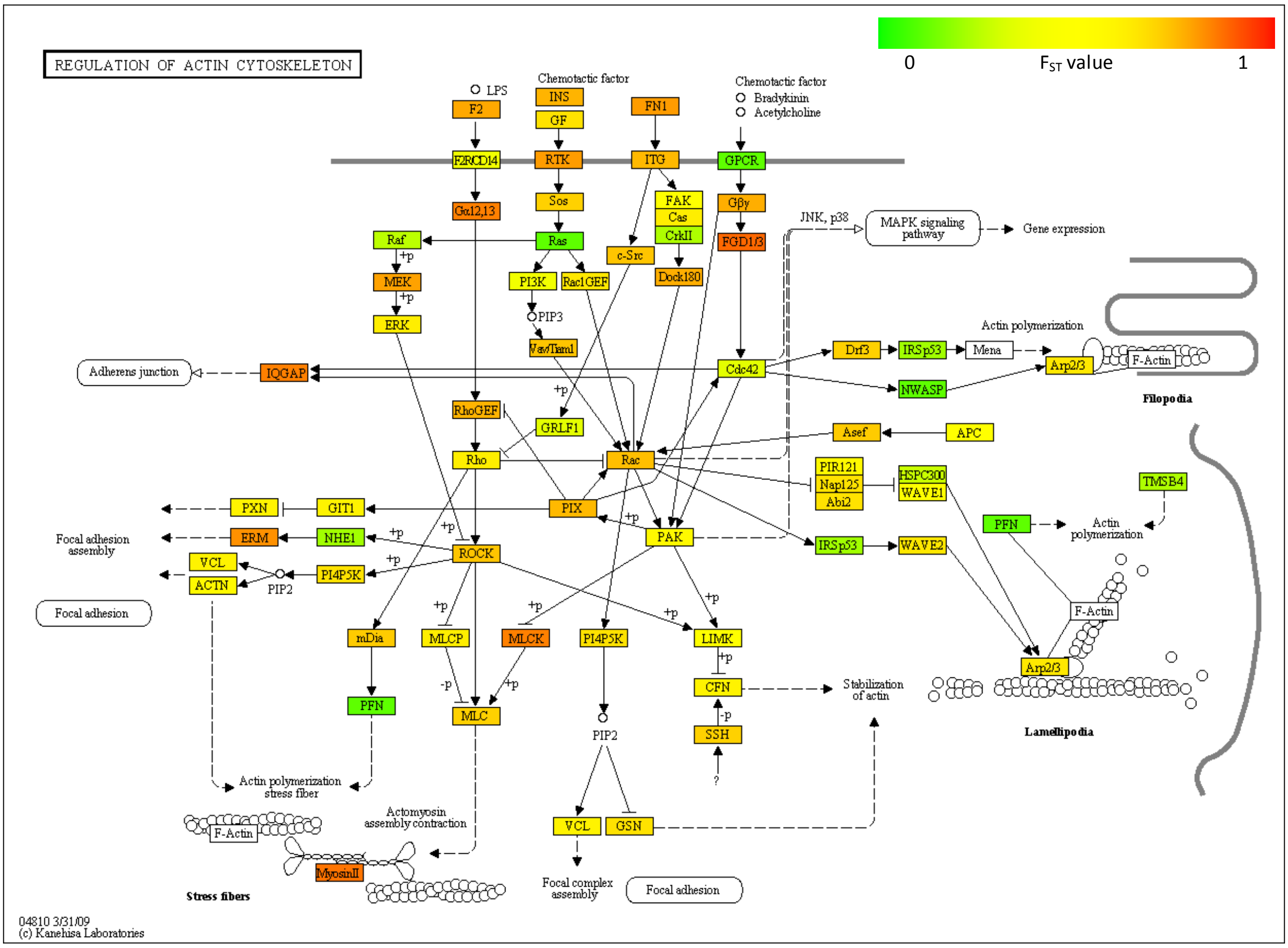

REGULATION OF ACTIN CYTOSKELETON
0
F_{ST} value
1
LPS
Chemotactic factor
Chemotactic factor
Bradykinin
Acetylcholine
F2
INS
GF
FN1
F2RCD14
RTK
ITG
GPCR
Gα12,13
Sos
FAK
Cas
CrkII
Gβγ
Raf
Ras
c-Src
FGD1/3
JNK, p38
MAPK signaling pathway
Gene expression
MEK
PI3K
Rac1GEF
Dock180
ERK
PIP3
Vav/Tiam1
Actin polymerization
Drf3
IRSp53
Mena
Arp2/3
F-Actin
Adherens junction
IQGAP
Cdc42
NWASP
Filopodia
RhoGEF
GRLF1
Asef
APC
Rho
Rac
PIR121
Nap125
Abi2
HSPC300
WAVE1
TMSB4
PXN
GIT1
PIX
PFN
Actin polymerization
Focal adhesion assembly
ERM
NHE1
ROCK
PAK
IRSp53
WAVE2
VCL
PI4P5K
ACTN
PIP2
F-Actin
Focal adhesion
mDia
MLCP
MLCK
PI4P5K
LIMK
Arp2/3
CFN
Stabilization of actin
PFN
MLC
SSH
PIP2
Lamellipodia
Actin polymerization stress fiber
Actomyosin assembly contraction
VCL
GSN
F-Actin
MyosinII
Stress fibers
Focal complex assembly
Focal adhesion
04810 3/31/09
(c) Kanehisa Laboratories

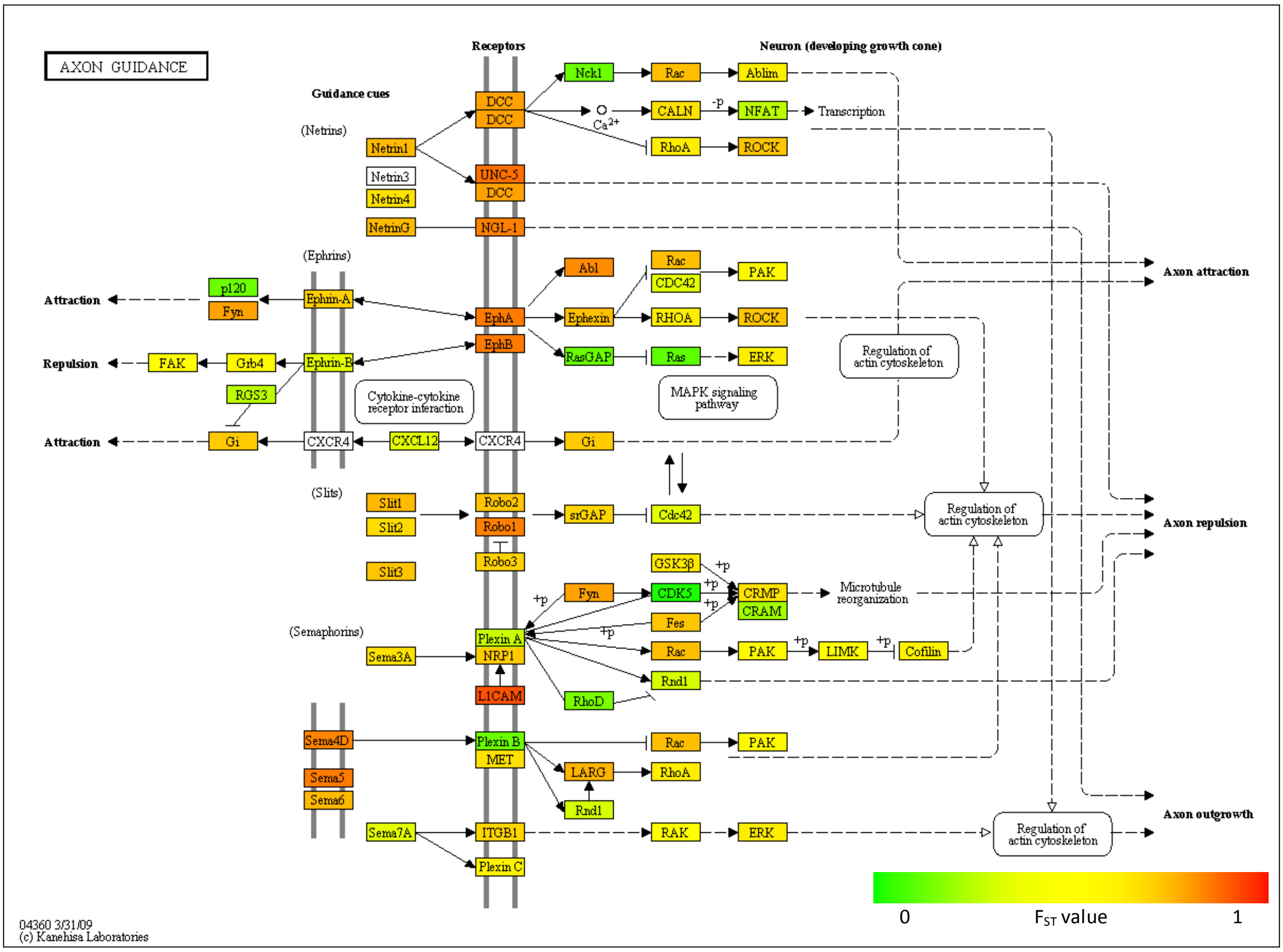
AXON GUIDANCE
Receptors
Neuron (developing growth cone)
Guidance cues
(Netrins)
Netrin1
Netrin3
Netrin4
NetrinG
DCC
DCC
UNC-5
DCC
NGL-1
Nck1
Rac
Ablim
CALN
NFAT
Transcription
Ca2+
RhoA
ROCK
(Ephrins)
Attraction
p120
Fyn
Ephrin-A
EphA
EphB
Abl
Rac
CDC42
PAK
Ephexin
RHOA
ROCK
RasGAP
Ras
ERK
Repulsion
FAK
Grb4
Ephrin-B
RGS3
Cytokine-cytokine receptor interaction
MAPK signaling pathway
Regulation of actin cytoskeleton
Attraction
Gi
CXCR4
CXCL12
CXCR4
Gi
Axon attraction
(Slits)
Slit1
Slit2
Slit3
Robo2
Robo1
Robo3
srGAP
Cdc42
Regulation of actin cytoskeleton
Axon repulsion
GSK3β
Fyn
CDK5
CRMP
CRAM
Microtubule reorganization
Fes
(Semaphorins)
Sema3A
Plexin A
NRP1
L1CAM
Rac
PAK
LIMK
Cofilin
Rnd1
RhoD
Sema4D
Sema5
Sema6
Plexin B
MET
Rac
PAK
LARG
RhoA
Rnd1
Sema7A
ITGB1
Plexin C
RAK
ERK
Regulation of actin cytoskeleton
Axon outgrowth
0
FST value
1
04360 3/31/09
(c) Kanehisa Laboratories

Antigen processing and presentation pathway

Gene	F_{ST}
HLA-DRB5	0.000
PSME1	0.011
HLA-DRB1	0.026
KLRC3	0.044
HLA-DMA	0.048
CTSL1	0.056
NFYB	0.061
HLA-E	0.070
HLA-B	0.082
IFNA6	0.089
LTA	0.092
HLA-DRA	0.093
KIR3DL2	0.095
IFNA14	0.096
HLA-A	0.098
HLA-C	0.100
IFNA13	0.102
CD74	0.103
HLA-DQB1	0.103
IFNA8	0.109
IFNA10	0.113
HLA-F	0.114
NFYA	0.114
IFNA1	0.122
RFX5	0.127
HSPA5	0.137
IFNA5	0.145
RFXANK	0.145
IFNA17	0.147
IFNA4	0.147
HLA-DMB	0.149
KIR2DL4	0.151
B2M	0.154
TAP2	0.155
IFNA16	0.170
CD4	0.176
IFI30	0.182
KLRC4	0.186
CANX	0.193
TAPBP	0.193
TAP1	0.194
KIR2DS4	0.197
RFXAP	0.198
HLA-DOA	0.203
CTSS	0.205

Calcium signaling pathway

Gene	F_{ST}
NTSR1	0.740
ADCY9	0.725
MYLK	0.660
ERBB4	0.650
CACNA1I	0.627
ADCY8	0.615
EDNRA	0.607
CHRM3	0.603
ADCY2	0.594
PHKA1	0.585
CACNA1A	0.574
EGFR	0.572
TRHR	0.570
ERBB2	0.562
GRM1	0.559
PLCD4	0.559
RYR3	0.556
CAMK2D	0.554
PPID	0.554
PPP3CA	0.553
PLCB1	0.545
GNA14	0.543
CACNA1G	0.539
CHRM5	0.539
PRKCB1	0.528
CACNA1D	0.518
ITPR3	0.516
CYSLTR1	0.514
AGTR1	0.509
SLC8A1	0.496
GRM5	0.494
ITPR1	0.493
PHKA2	0.491
ATP2B4	0.482
GNAS	0.482
SLC8A2	0.479
ATP2B3	0.476
CACNA1C	0.474
PLCZ1	0.472
CACNA1F	0.469
OXTR	0.468
GRIN2A	0.467
PLCB4	0.465
CHRM2	0.462
HTR5A	0.462
ADORA2B	0.459
PLCG2	0.457
ITPR2	0.455
RYR2	0.451
ATP2A2	0.450
LHCGR	0.447
PRKX	0.446
CACNA1B	0.445
CCKBR	0.445
MYLK2	0.444
CHRNA7	0.443
CALM2	0.442
PPP3CB	0.440
TACR3	0.440
CACNA1S	0.439
GNAL	0.436
TRPC1	0.434
CALM1	0.431
GNAQ	0.423
ADRA1A	0.422
ADCY7	0.421
CACNA1E	0.421
NOS1	0.421
SLC8A3	0.421
ATP2B2	0.420
CD38	0.418
HTR2C	0.418

Focal adhesion pathway

Gene	F_{ST}	Gene	F_{ST}	Gene	F_{ST}
VAV2	0.935	FYN	0.556	DIAPH1	0.449
GRB2	0.752	MAP2K1	0.554	PAK2	0.449
VEGFC	0.732	SRC	0.550	BRAF	0.445
PARVA	0.718	ITGA6	0.542	MYLK2	0.444
TNXB	0.715	VWF	0.534	RELN	0.443
COL11A1	0.694	PRKCB1	0.528	COL4A2	0.442
LAMA5	0.670	DOCK1	0.527	ITGB6	0.440
VAV1	0.667	ACTN2	0.504	AKT1	0.439
MYLK	0.660	ITGA11	0.502	SOS1	0.434
ACTN1	0.653	LAMA3	0.501	COL4A6	0.430
PPP1CA	0.629	TLN2	0.496	ITGA1	0.429
LAMB4	0.620	PDGFD	0.492	PIK3CA	0.429
TNN	0.589	PAK7	0.484	MYL9	0.427
COL5A1	0.588	PIK3CD	0.483	LAMA4	0.424
IGF1R	0.584	FLT1	0.482	PDGFC	0.423
LAMA1	0.584	ITGAV	0.481	COL1A2	0.422
FLNB	0.574	PAK3	0.478	ITGA8	0.422
EGFR	0.572	RAC1	0.478	ITGB1	0.421
LAMA2	0.571	CAV1	0.473	PARVB	0.421
RAF1	0.571	CCND1	0.469	CAPN2	0.420
FN1	0.569	ROCK1	0.468	HGF	0.419
FLNA	0.567	ITGA9	0.464	PPP1CC	0.418
SHC3	0.565	MAPK10	0.463	LAMC3	0.417
ERBB2	0.562	VAV3	0.460		
ITGA7	0.559	COL5A2	0.458		
		COL6A6	0.452		

ECM-receptor interaction pathway

Gene	F_{ST}
TNXB	0.715
COL11A1	0.694
SV2B	0.692
LAMA5	0.670
LAMB4	0.620
TNN	0.589
COL5A1	0.588
LAMA1	0.584
SDC2	0.576
LAMA2	0.571
FN1	0.569
ITGA7	0.559
SV2C	0.545
ITGA6	0.542
VWF	0.534
DAG1	0.515
ITGA11	0.502
LAMA3	0.501
SDC4	0.488
ITGAV	0.481
SV2A	0.475
ITGA9	0.464
COL5A2	0.458
CD44	0.454
COL6A6	0.452
RELN	0.443
COL4A2	0.442
ITGB6	0.440
HSPG2	0.436
COL4A6	0.430
ITGA1	0.429
LAMA4	0.424
COL1A2	0.422
ITGA8	0.422
ITGB1	0.421
LAMC3	0.417

Adherens junction pathway

Gene	F_{ST}
PARD3	0.682
CTNNA3	0.677
ACTN1	0.653
TCF7L2	0.647
TCF7L1	0.626
PTPRM	0.616
IGF1R	0.584
INSR	0.579

EGFR	0.572
ERBB2	0.562
FYN	0.556
SRC	0.550
FER	0.543
CTNNA2	0.538
PVRL3	0.527
ACTN2	0.504
PTPRJ	0.502

LEF1	0.501
SMAD3	0.487
TGFBR1	0.480
RAC1	0.478
SNAI1	0.474
PVRL1	0.462
IQGAP1	0.460

Regulation of actin cytoskeleton pathway

Gene	F_{ST}
VAV2	0.935
MSN	0.930
FGD1	0.711
MYH9	0.693
ITGAE	0.692
ARPC1B	0.688
VAV1	0.667
MYLK	0.660
ACTN1	0.653
GNA13	0.651
IQGAP2	0.651
ARHGEF1	0.641
SSH3	0.630
PPP1CA	0.629
EZR	0.613
CHRM3	0.603
PIP5K3	0.581
EGFR	0.572
FGF2	0.571
RAF1	0.571
FN1	0.569
ITGA7	0.559
MAP2K1	0.554
F2	0.546
ITGA6	0.542
CHRM5	0.539
FGFR2	0.538
FGF14	0.533
TIAM1	0.532
DOCK1	0.527
PIP4K2A	0.521
DIAPH2	0.519
GNG12	0.519
ARHGEF12	0.518
FGF12	0.515
INS	0.515
ITGAM	0.515
ACTN2	0.504
ARHGEF7	0.502
ITGA11	0.502
SSH2	0.496
PAK7	0.484
PIK3CD	0.483
ITGAV	0.481
PAK3	0.478
RAC1	0.478
KRAS	0.471
ROCK1	0.468
CSK	0.464
ITGA9	0.464
CHRM2	0.462
IQGAP1	0.460
VAV3	0.460
ARHGEF4	0.455
DIAPH1	0.449
PAK2	0.449
BRAF	0.445
MYLK2	0.444
ITGB6	0.440
ITGAX	0.439
LIMK2	0.437
SSH1	0.436
SOS1	0.434
ITGA1	0.429
PIK3CA	0.429
MYL9	0.427
DIAPH3	0.425
FGF19	0.424
ITGA8	0.422
ITGB1	0.421
GNA12	0.420
ARPC2	0.419
FGFR3	0.419
PPP1CC	0.418
APC	0.416
ITGB2	0.414
CRKL	0.413
PAK6	0.411

Axon guidance pathway

Gene	F_{ST}
EPHA6	0.817
L1CAM	0.788
PLXNA3	0.724
UNC5D	0.697
SEMA5B	0.677
EPHB1	0.667
EPHA2	0.663
LRRC4C	0.661
ROBO1	0.661
UNC5A	0.660
SEMA4C	0.655
SEMA6D	0.654
SEMA3E	0.648
SEMA4F	0.634
ABL1	0.619
SEMA3F	0.618
EPHB6	0.597
DCC	0.559
GNAI2	0.558
FYN	0.556
PPP3CA	0.553
EPHB2	0.545
SEMA3B	0.544
SEMA6A	0.531
UNC5C	0.529
ARHGEF12	0.518
NFATC1	0.518
EFNA5	0.511
NTNG1	0.502
ABLIM2	0.501
SLIT1	0.498
NTN1	0.492
SEMA6C	0.489
ABLIM1	0.486
PAK7	0.484
PAK3	0.478
RAC1	0.478
SEMA5A	0.475
NGEF	0.473
KRAS	0.471
ROCK1	0.468
SLIT3	0.468
NFATC3	0.467
FES	0.465
SEMA3D	0.464
EFNA4	0.463
ROBO2	0.461
PAK2	0.449
GNAI1	0.446
NRP1	0.443
PPP3CB	0.440
LIMK2	0.437
NFATC2	0.437
ROBO3	0.437
SEMA4B	0.436
UNC5B	0.436
EFNA1	0.430
SEMA3C	0.428
ITGB1	0.421
SRGAP2	0.418
EFNB2	0.414
EPHA4	0.411
PAK6	0.411
SRGAP3	0.408

GAD diseases classification

Diseases classification as present in the Genetic Association Database (http://geneticassociationdb.nih.gov/).

Class	Disease
AGING	
	age
	age of menarche
	age-associated memory impairment
	ageing
	aging
	Alzheimer's Disease and Vascular Dementia
	anticholinergic challenge-induced memory impairment
	bone resorption
	Brain aging
	cerebral aging
	cerebral amyloid angiopathy
	cholesterol, HDL; blood pressure, arterial; stroke; longevity
	clinical parameters and endocrine status in elderly men
	cognitive decline
	cognitive function
	cognitive function in older men
	cognitive impairment
	cognitive performance
	degeneration of intervertebral discs
	disc degeneration, lumbar spine
	disc disease, intervertebral
	diurnal preference
	Dominant Progressive Deafness
	Early cognitive decline
	exceptional longevity
	Exercise-induced changes in insulin
	fat-free mass and sarcopenia
	harm avoidance behaviour in an elderly population.
	life expectancy
	Longevity
	lumbar disc disease
	memory decline
	memory disturbance
	memory impairment
	muscle strength
	muscle testing

	obesity
	Osteoarthritis
	osteoporosis
	physical function
	protein fraction levels
**CANCER**	
	5' CpG island hypermethylation
	acute leukemia
	acute myeloid leukemia
	adenocarcinoma of the lung
	adenocarcinoma of the upper gastrointestinal tract.
	adenocarcinomas of the parotid gland
	adenomatous polyposis
	adenomatous polyposis coli
	adult brain tumors
	adult T-cell leukemia
	advanced colorectal cancer
	advanced prostate cancer
	aerodigestive tract cancers
	aflatoxin-related hepatocarcinogenesis
	alpha-fetoprotein
	alpha-particle carcinogenesis
	anaplastic astrocytoma; glioblastoma multiforme
	angiomyolipomas, renal
	Barrett esophagus esophageal cancer
	betel-quid chewing
	bladder cancer
	bladder cancer, urinary
	bladder cancer; prostate cancer
	blast crisis of chronic myeloid leukaemia
	blastic crisis
	bone cancer; soft tissue sarcoma
	brain cancer
	brain tumor
	brain tumors
	breast and lung cancer
	breast cancer
	breast cancer breast cancer, male colorectal cancer ovarian cancer prostate cancer
	breast cancer by the age of 50 years
	breast cancer cervical cancer colorectal cancer esophageal cancer lung cancer stomach cancer
	breast cancer colorectal cancer stomach cancer
	breast cancer development
	breast cancer endometriosis uterine fibroids
	breast cancer melanoma
	breast cancer ovarian cancer

breast cancer recurrence
breast cancer risk
breast cancer risk and prenatal viability
breast cancer survival
breast cancer, male
breast cancer; colorectal cancer; esophageal cancer; stomach cancer
breast cancer; endometrial cancer
breast cancer; insulin-like growth factor
breast cancer; ovarian cancer
breast cancer; tamoxifen, prophylactic effect of
breast carcinoma
cancer
cancer lung cancer
Cancer progression and tumor cell motility
cancer susceptibility
cancer tissues
cancer.
cancer; lung cancer
cardia cancer stomach cancer
Cell Cancer
cervical cancer
cervical cancer endometrial cancer ovarian cancer
cervical cancer ovarian cancer
chemotherapy-induced leukemia
childhood brain tumor
Childhood Leukemia
cholangiocarcinoma
cholangiocarcinoma homocysteine
chronic atrophic gastritis and gastric carcinoma
cirrhosis, alcoholic; liver cancer
clinicopathological characteristics of hepatocellular carcinoma
clonal evolution of chronic myeloid leukemia
clonal homozygosity of rectal cell carcinoma
colon adenomas/carcinomas
colon cancer
colon polyps
colorectal adenocarcinomas
colorectal adenomas
colorectal cancer
colorectal cancer hyperplastic polyposis
colorectal cancer stomach cancer
colorectal cancer, nonpolyposis
colorectal cancer.
colorectal cancer; colon polyps
colorectal cancer; drug hypersensitivity
colorectal cancer; endometrial cancer
colorectal cancers

colorectal carcinomas
colorectal neoplasia.
colorectal polyps (adenoma or hyperplastic)
conventional renal cell carcinoma
C-peptide insulin-like growth factor sex hormones
cryptorchidism infertility, male testicular cancer
cutaneous malignant melanoma
cutaneous malignant melanoma.
cutaneous melanoma which is largely independent of skin type and hair color
CYP1A1 genetic polymorphisms
CYP1A1 polymorphisms
decreased lung cancer risk
decreased risk for breast cancer by age 50
developing lung cancer
diet lifestyle and risk of colon cancer
diffuse gastric cancer
diffuse large B-cell lymphoma
early-onset prostate cancer
early-stage breast cancers
endometrial cancer
endometrial cancer risk.
endometrial carcinoma
endometrioid and clear cell ovarian cancer
esophageal cancer
esophageal cancer and gastric cardiac carcinoma
esophageal cancer gastric cardiac adenocarcinoma
esophageal cancer gastric cardiac cancer
esophageal cancer noncardia gastric carcinoma stomach cancer
esophageal cancer stomach cancer
esophageal cancer; cardiac cancer
esophageal cancer; gastric cardia cancer
esophageal cancer; gastric cardiac cancer
esophageal cancer; stomach cancer
esophageal carcinoma
esophageal squamous cell carcinoma
estrogen receptor status
extra-adrenal and/or malignant phaeochromocytomas
familial adenomatous polyposis.
familial melanoma
familial or sporadic prostate cancer.
fibromatosis, aggressive
fibrosis, subcutaneous
folate pool
follicular lymphoma
follicular lymphoma to diffuse large-cell lymphoma
gangliogliomas
gastric adenocarcinoma

gastric cancer
gastric carcinoma
gastric carcinoma risk
gastric cardia adenocarcinoma
gastritis, chronic atrophic; stomach cancer
gastroesophageal cancer
gene polymorphism coding for increased IL-10 production
genetic polymorphism
germline BRCA1 mutation
glioblastoma multiforme
H. pylori infection stomach cancer
H. pylori infection; stomach cancer
Haematological Neoplasias
head and neck cancer
head and neck cancer lung cancer
head and neck cancer mutagen sensitivity
hepatitis B liver cancer
hepatitis B; cirrhosis; liver cancer
hepatitis C; liver cancer
hepatocellular carcinoma
hepatocellular carcinomas
hereditary and sporadic prostate cancer risk.
hereditary and sporadic prostate cancer susceptibility.
hereditary non-polyposis colon cancer.
HIF-1alpha protein expression
high exposure to aflatoxin B1.
high-grade soft tissue sarcoma
Hodgkin's disease
Hodgkin's disease leukemia, myeloid multiple myeloma myelodysplastic syndrome
H-ras allele loss
human long-term memory
hypopharyngeal cancer laryngeal cancer
increased aggressiveness
increased chromosome aberrations and higher lung cancer risk
increased development of adult T-cell leukemia/lymphoma
increased incidence of invasive breast cancer
increased lung cancer risk
increasing risk and a poor prognosis of renal cell carcinoma
kidney angiomyolipomas
kidney cancer
kidney cancer; bladder cancer
laryngeal cancer
laryngeal squamous cell carcinoma
leiomyoma
leukemia
leukemia, acute lymphoblastic

leukemia, acute myeloid
leukemia, chronic lymphocytic
leukemia, chronic myelogenous
leukemia, lymphoid
leukemia, myeloid
leukemia/lymphoma, T-Cell
leukemia; leukemia, myelodysplastic (TRLIMDS)
leukemia; Noonan syndrome
liver cancer
liver cancer; liver disease
lung adenocarcinoma
lung cancer
lung cancer and preneoplastic bronchial lesions
lung cancer risk
lung cancer smoking behavior
lung cancer susceptibility and prognosis
lung cancer, non-small cell
lung cancer; breast cancer
lung cancer; breast cancer; bladder cancer; cervical cancer
lung cancer; breast cancer; liver cancer; oral cancer; thyroid cancer; cervical cancer
lung cancer; colorectal cancer; esophageal cancer; head and neck cancer
lung cancer; esophageal cancer
lung cancer; esophageal cancer; stomach cancer; bladder cancer
lung cancer; Lambert-Eaton myasthenic syndrome
lung carcinoma
Lung carcinomas
lung squamous cell carcinoma
lymph node involvement and other histopathological indicators of high metastatic potential
lymphocytic leukemia
lymphoma
lymphoma, cutaneous T-cell
lymphoma, non-Hodgkin
lymphovascular tumor invasion stomach cancer
macroglobulinemia
male breast cancer
malignancy
malignant melanoma
mamographic density
melanoma
melanoma skin cancer
melanoma, cutaneous
meningioma
metastases
metastatic breast cancer
metastatic colorectal cancer

	Metastatic Melanoma
	metastatic progression and poor survival
	microsatellite instability
	MPO -463 A allele
	mucosa-associated lymphoid tissue lymphoma
	multiple endocrine neoplasia
	multiple endocrine neoplasia type 2A
	Multiple familial trichoepithelioma and familial cylindromatosis
	multiple myeloma
	multiple oesophageal dysplasia
	myeloblastic leukemias
	myeloma, multiple
	nasopharyngeal cancer
	nasopharyngeal carcinoma
	neuroblastoma
	neurofibromatosis 1
	Neurofibromatosis type 2
	neurofibromatosis2
	nevoid basal cell carcinoma syndrome associated
	noncardia gastric cancer
	Non-Hodgkin's B cell lymphoma
	non-Hodgkin's lymphoma
	non-papillary renal cell carcinomas
	non-small cell lung cancer
	oesophageal squamous cell carcinoma
	oligodendrogliomas
	oral cancer
	oral cancer oral submucous fibrosis
	oral cancer; oral submucous fibrosis
	oral clefting
	oral squamous cell cancer
	orolaryngeal cancer
	orolaryngeal carcinoma
	osteosarcoma
	ovarian cancer
	ovarian cancer; thyroid cancer; pheochromocytoma
	ovarian carcinoma
	pancreatic adenocarcinoma
	pancreatic cancer
	pancreatic cancer; pancreatitis, chronic
	pancreatic carcinoma
	pancreatitis; pancreatic cancer
	paragangliomas
	paragangliomas, head and neck
	paragangliomas, head and neck; pheochromocytomas
	parathyroid cancer
	peptic ulcer stomach cancer

pheochromocytoma
pituitary cancer
predisposition to cancer
prenatal viability
progression of a prostate carcinoma
prostate cancer
prostate cancer prostate specific antigen
prostate carcinoma
prostate enlargement
prostatic hyperplasia
prostatic hyperplasia; prostate cancer
protein loss
proximal chromosome 9p to q and distal chromosome 9q
PSA mRNA expression
radiation, ionizing, sensitivity to
radiation-induced damage to normal tissues
reduced lung cancer risk and a modulating effect on nucleotide excision repair capacity
reduced risk of lung cancer
renal cancer
renal cell carcinoma
retinoblastoma
rhabdoid tumors
schistosomiasis
sensitivity to TZT-1027
skin cancer, nonmelanoma
skin cancer, non-melanoma
skin cancer; squamous cell carcinoma; carcinoma, basal cell
skin carcinomas
smoking
soft tissue sarcoma
squamous cell carcinoma
squamous cell carcinoma of the head and neck
stomach cancer
stomach cancer vascular endothelial growth factor levels
stomach cancer; gastric ulcer; gastritis; duodenal ulcer
stomach cancer; gastritis
stomach cancer; ovarian cancer
stomach cancer; stomach atrophy
testicular cancer
thyroid cancer
thyroid cancer; Hirschsprung's disease
thyroid cancer; thyroid carcinoma, sporadic medullary
thyroid carcinoma
transcriptional inactivation of the FHIT gene
transitional cell carcinoma of the bladder.
tuberculosis; esophageal cancer

	tumor progression
	tumor progression of BCR/ABL negative chronic myeloproliferative disorders
	tumor proliferation and other prognostic indicators
	tumour grade
	tumour kinetics and chromosomal instability
	tumour progression
	upper aerodigestive tract cancer
	urinary bladder cancer
	urothelial cancer
	uterine endometrial carcinoma
	von Hippel-Lindau syndrome (VHL).
	von Hippel-Lindau tumour
	von Hippel-Lindau type 2A
	vulvar cancer
	vulvar carcinogenesis
	Wilm's tumor
	wilms' tumor and congenital male genitourinary malformation
	Wilms tumors
**CARDIOVASCULAR**	
	Abdominal adiposity and Hypertension
	abdominal aortic aneurysm
	abdominal aortic aneurysm homocysteine hypertension
	acute coronary syndrome
	acute coronary syndrome diabetes, type 2 hypertension
	acute myocardial infarction
	age at first coronary bypass operation
	aging
	amyloid cardiomyopathy
	aneurysm, abdominal aortic
	aneurysm, intracranial
	aneurysmal subarachnoid hemorrhage
	angina
	angina, unstable
	angina, vasospastic
	angina, vasospastic atherosclerosis, coronary
	angioedema
	angiotensin-converting enzyme genotype
	aortic dissection
	aortic dissection atherosclerosis, coronary stroke
	aortic stenosis
	aortic stiffness and disease severity
	aortoarteritis
	arrhythmia, cardiac
	arterial disease
	arterial stiffness
	arterial wall changes

arterial wall thickness
arteriovenous dysplasias brain hemorrhage
arteriovenous fistulas
atherosclerosis
atherosclerosis diabetes, type 2 inflammation zinc
atherosclerosis, carotid
atherosclerosis, carotid coronary artery calcium, intimal medial thickness
atherosclerosis, carotid intima media thickness
atherosclerosis, carotid kidney failure, chronic
atherosclerosis, carotid myocardial infarct
atherosclerosis, coronary
atherosclerosis, coronary body mass cholesterol, HDL cholesterol, LDL cholesterol, VLDL
atherosclerosis, coronary carotid artery damage
atherosclerosis, coronary cholesterol
atherosclerosis, coronary cholesterol, HDL
atherosclerosis, coronary cholesterol, HDL triglycerides
atherosclerosis, coronary C-reactive protein myocardial infarct
atherosclerosis, coronary endothelial function hypercholesterolemia lipids
atherosclerosis, coronary lipids lipoprotein
atherosclerosis, coronary lipoprotein
atherosclerosis, coronary lipoprotein triglycerides
atherosclerosis, coronary myocardial infarct
atherosclerosis, coronary unstable coronary syndrome
atherosclerosis, coronary; acute coronary syndrome
atherosclerosis, coronary; diabetes, type 1; nephropathy in other diseases
atherosclerosis, coronary; diabetes, type 2
atherosclerosis, coronary; diabetes, type 2; C-reactive protein
atherosclerosis, coronary; diabetes, type 2; lipoprotein
atherosclerosis, coronary; hematology indices
atherosclerosis, coronary; homocysteine
atherosclerosis, coronary; hypertension
atherosclerosis, coronary; lipid metabolism disorders
atherosclerosis, coronary; lipids; longevity; apoB levels
atherosclerosis, coronary; lipoprotein
atherosclerosis, coronary; lipoprotein; lipids
atherosclerosis, coronary; metabolic syndrome
atherosclerosis, coronary; metabolism disorders
atherosclerosis, coronary; myocardial dysfunction, non-ischaemic
atherosclerosis, diabetic
atherosclerosis, generalized
atherosclerosis, generalized body fat
atherosclerosis, generalized cardiovascular disease cerebrovascular disease myocardial infarct stroke, ischemic
atherosclerosis, generalized cholesterol, HDL triglycerides
atherosclerosis, generalized C-reactive protein
atherosclerosis, generalized myocardial infarct

	atherosclerosis, generalized; vascular disease
	atherosclerosis; angina
	atherosclerosis; coronary vascular endothelial dysfunction
	Atherothrombosis
	atherothrombotic disease
	atrial fibrillation
	bilateral iliac vein thrombosis
	blood flow
	blood pressure
	blood pressure phenotypes.
	blood pressure, arterial
	blood pressure, arterial body mass cholesterol, HDL metabolic syndrome
	blood pressure, arterial body mass glucose insulin lipids
	blood pressure, arterial body mass glucose tolerance lipids
	blood pressure, arterial body mass kidney disease metabolic syndrome myocardial infarct obesity triglycerides
	blood pressure, arterial cholesterol
	blood pressure, arterial cholesterol insulin resistance metabolic syndrome
	blood pressure, arterial diabetes, type 2 glucose insulin
	blood pressure, arterial diabetes, type 2 insulin
	blood pressure, arterial electrolyte levels
	blood pressure, arterial hypertension
	blood pressure, arterial hypertriglyceridemia
	blood pressure, arterial intima-media thickness
	blood pressure, arterial kidney function
	blood pressure, arterial obesity
	blood pressure, arterial urinary sodium
	blood pressure, arterial; arterial wall changes
	blood pressure, arterial; atherosclerosis
	blood pressure, arterial; carotid atherosclerosis
	blood pressure, arterial; cystic fibrosis
	blood pressure, arterial; renin activity; aldosterone
	blood pressure; hyperlipidemia, familial combined
	body mass
	body mass cardiovascular risk factors lipoprotein
	body mass coronary artery bypass graft
	body mass; left ventricular function; blood pressure; left ventricular structure
	brain aneurysm
	brain function
	brain hemorrhage
	brain hemorrhage cerebral infarct, atherothrombotic stroke, lacunar
	brain hemorrhage; stroke, ischemic
	brain hemorrhage; stroke; stroke, lacunar
	brain infarction
	Brugada syndrome
	Budd-Chiari syndrome hematology indices polycythemia vera
	Budd-Chiari syndrome liver transplant portal vein thrombosis

calcific aortic valve stenosis
calcium cardiac death heart disease, ischemic myocardial infarct phosphate
cardiac and cutaneous amyloidosis
cardiac arrhythmias and sudden death
cardiac conduction disturbances and degenerative changes
cardiac death
cardiac death cardiovascular disease oxidative stress
cardiac death myocardial infarct
cardiac death QT interval
cardiac death; cardiac morbidity
cardiac function
cardiac growth
cardiac repolarization
cardiac sarcoidosis
cardiomyopathy
cardiomyopathy hypertension left ventricular hypertrophy
cardiomyopathy, idiopathic dilated
cardiomyopathy, nonfamilial hypertrophic
cardiomyopathy; heart failure
cardiomyopathy; MELAS syndrome
cardiopulmonary disease
cardiorespiratory fitness cholesterol, HDL
cardiotoxicity, anthracycline-induced
cardiovascular
cardiovascular abnormalities
cardiovascular complications diabetes, type 2 hypertension obesity polycystic ovarian syndrome
cardiovascular death
cardiovascular disease
cardiovascular disease cholesterol cholesterol, HDL cholesterol, VLDL hyperlipidemia intimia-media thickness lipoprotein triglycerides
cardiovascular disease cholesterol, LDL diabetes, type 2 triglycerides
cardiovascular disease; bilirubin
cardiovascular disease; carotid artery damage
cardiovascular disease; cerebrovascular disease
cardiovascular disease; diabetes, type 1; proteinuria
cardiovascular disease; kidney failure, chronic
cardiovascular disease; stroke; stroke, ischemic
cardiovascular events in non-smokers
cardiovascular mortality
cardiovascular response to metoprolol
cardiovascular risk
cardiovascular risk factors
carotid and femoral artery stiffness
carotid arterial wall thickness
carotid artery atherosclerosis
carotid artery damage

carotid artery distensibility
carotid artery intima-media thickness
carotid artery stenosis
carotid atherosclerosis
carotid intimal-medial thickening
carotid intima-media thickness
carotid wall thickening
cerebral amyloid angiopathy
cerebral amyloid angiopathy-related hemorrhage.
cerebral arteriopathy
cerebral atherosclerosis
cerebral circulation
Cerebral Circulation in Smokers
cerebral hemorrhage
cerebral infarct
cerebral infarct, atherosclerotic cholesterol, HDL intima media thickness triglycerides
cerebral infarct, atherothrombotic
cerebral infarct; stroke, lacunar
cerebral infarction
cerebral ischemia
cerebral venous thrombosis
cerebrovascular disease
cerebrovascular disease, ischemic
cerebrovascular disease, ischemic; fibrinogen
cervical artery dissection, spontaneous
cholesterol cholesterol, HDL cholesterol, LDL fatty acid glucose insulin lipoprotein triacylglycerols
cholesterol cholesterol, HDL cholesterol, LDL lipoprotein triglycerides
cholesterol lipoprotein retinyl palmitate triglycerides
cholesterol triglycerides
cholesterol, HDL
cholesterol, HDL triglycerides
cholesterol, HDL; triglycerides; coronary artery disease; cerebral infarction
Cigarette Smoking. Coronary Artery Disease. and Diabetes
congenital cardiac malformations
congestive heart failure
coronary artery calcification
coronary artery calcium
coronary artery disease
coronary artery disease and myocardial infarction
coronary artery disease, occlusive
coronary artery disease; aortic stiffness
coronary artery disease; coronary atherosclerosis
coronary artery disease; P-selectin
coronary artery disease; stroke
coronary artery disease; sudden cardiac death

	coronary artery ectasia
	coronary artery luminal dimensions
	coronary artery plaque calcification
	coronary artery spasm
	coronary artery stenosis
	coronary artery vasoconstriction.
	coronary atherosclerosis
	coronary atherosclerosis.
	coronary atherosclerotic heart disease in Chinese
	coronary calcification
	coronary endothelial vasodilator function
	coronary events
	Coronary Heart Disease
	coronary heart disease among Japanese.
	coronary heart disease and serum very low density lipopro
	coronary heart disease in non-insulin-dependent diabetic
	coronary heart disease in younger individuals.
	coronary heart disease, transplant associated
	coronary heart disease.
	coronary heart disease; hypertension; myocardial infarction
	coronary heart disease; myocardial infarction
	coronary heart disease; myocardial infarction; stroke; cerebrovascular disease
	coronary in-stent restenosis.
	coronary spasm.
	coronary spastic angina.
	coronary vasculopathy
	coronary vasomotor function
	CP infection.
	C-reactive protein
	C-reactive protein myocardial infarct
	decreased blood pressure and plasma triglycerides
	deep venous thrombosis
	dementia
	development of coronary artery lesions
	diabetes, type 2 dyslipidemia heart disease, ischemic
	diabetes, type 2 hypertension lipoprotein
	diabetes, type 2; hypertension
	diabetes, type 2; hypertension; atherosclerosis; cerebrovascular disease; dyslipemia
	diastolic blood pressure
	dilated cardiomyopathy
	early onset ischemic heart disease.
	early-onset aggressive diffuse amyloidosis
	EKG, abnormal
	emphysema
	endothelial dysfunction hypertension
	endothelial dysfunction in normal humans.

	endothelial function
	endothelial function von Willebrand factor
	endothelium-dependent arterial dilation
	epinephrine lung function norepinephrine
	Essential Hypertension
	expired myocardial infarction
	familial combined hyperlipidemia
	familial hypertension of early onset.
	familial hypertrophic cardiomyopathy.
	familial premature myocardial infarction.
	ferritin
	fetal loss, late
	fibrinogen myocardial infarct
	flow-mediated dilatation of the brachial artery
	gastric cancer
	giant cell arteritis
	giant cell arteritis.
	giant cell arteritis; polymyalgia rheumatica
	grade II hypertension
	heart anomalies, congenital
	heart disease risk factors hypertension leptin obesity
	heart disease, ischemic
	heart disease, ischemic stroke, ischemic
	heart disease, ischemic; atherosclerosis, coronary; hyperlipidemia
	heart disease, ischemic; cerebrovascular disease, ischemic
	heart disease, ischemic; insulin
	heart disease, ischemic; peripheral vascular disease
	heart failure
	heart period signal
	heart rate
	heart rate variability
	heart rate, resting
	heart rate; arrhythmia, cardiac
	heart rate; risperidone metabolism
	heart transplant
	heart transplant complications
	Heart-Hand Syndrome, Holt-Oram Syndrome
	hemorrhage, intracerebral
	hemorrhagic and ischemic stroke
	high coronary heart disease risk particularly affects ser
	high density lipoprotein cholesterol level
	hypercholesterolemia coronary artery disease
	hypercholesterolemia peripheral vascular disease
	hyperhomocysteinemia and cardiovascular disease
	hypertension
	hypertension and left ventricular mass
	hypertension and obesity

hypertension NADPH oxidase activity
hypertension vascular aging
hypertension, cirrhotic portal
hypertension, pregnancy induced
hypertension, pregnancy induced preeclampsia
hypertension, pulmonary arterial
hypertension,response to exercise
hypertension; autonomic nervous system dysfunction
hypertension; blood pressure
hypertension; blood pressure, arterial
hypertension; cardiovascular disease
hypertension; cerebral infarction
hypertension; cirrhosis
hypertension; exercise
hypertension; glucose
hypertension; insulin; metabolic syndrome; hypertriglyceridemia
hypertension; insulin; obesity
hypertension; left ventricular hypertrophy
hypertension; left ventricular structure
hypertension; myocardial infarction
hypertension; preeclampsia
hypertension; renal disease, end stage
hypertension; renal sodium handling
hypertension; stroke
hypertension; stroke, ischemic
hypertension; stroke, lacunar; atherothrombotic brain infarction
hypertrophic cardiomyopathy
hypertrophic cardiomyopathy and cytochrome c oxidase deficiency
idiopathic deep venous thrombosis
idiopathic pulmonary fibrosis
increased cardiopulmonary morbidity
increased vascular reactivity
inherited cardiac arrhythmia long QT syndrome
intima media thickness
intima-media thickness
intima-media thickness myocardial infarct
intima-media thickness, carotid
intima-media thickness; flow-mediated dilation of the brachial artery
intracranial aneurysms
intracranial hemorrhage; white matter disease
ischemia
kawasaki disease
left ventricular dysfunction
left ventricular hypertrophy
left ventricular hypertrophy; blood pressure, arterial
left ventricular mass
left ventricular mass ventricular remodeling

left ventricular structure
lipids; myocardial infarction
lipoprotein triglycerides
long QT syndrome
longevity
long-QT syndrome
matrix metalloproteinase-3 concentration myocardial infarct
metabolic syndrome
microangiopathy, thrombotic
microvascular function
mitral valve prolapse
mitral valve prolapse.
myocardial infarct
myocardial infarct stroke, ischemic
myocardial infarct, mortality in
myocardial infarct; atherosclerosis
myocardial infarct; atherosclerosis, coronary
myocardial infarct; atherosclerosis, coronary; C-reactive protein
myocardial infarct; cardiac death
myocardial infarct; cardiovascular disease
myocardial infarct; carotid intima-media thickness
myocardial infarct; cerebrovascular infart
myocardial infarct; diabetes, type 2
myocardial infarct; heart disease, ischemic
myocardial infarct; heart disease, ischemic; atherosclerosis, coronary
myocardial infarct; heart failure
myocardial infarct; kidney disease
myocardial infarct; lipoprotein; cerebral infarct
myocardial infarct; stroke, atherothrombotic
myocardial infarct; stroke; angina; peripheral vascular disease
myocardial infarct; thrombosis, deep vein
myocardial infarct; triglycerides
myocardial infarction
myocardial infarction and stroke
myocardial infarction; angina
myocardial infarction; stable angina of effort
myocardial infarction; stroke
myocardial infarction; sudden cardiac death
myocardial injury
myocardial ischemia
neuropathy
nonfamilial idiopathic dilated cardiomyopathy
nonfatal acute myocardial infarction
nonfatal myocardial infarction.
non-ST-elevation acute coronary syndromes
obesity
oxidized low-density lipoprotein and cardiolipin autoantibodies

paraoxinase 1 activity
peripheral arterial disease
peripheral demyelinating neuropathies
peripheral vascular disease
premature coronary artery disease
premature coronary artery disease and familial hypoalphalipoproteinemia
premature coronary artery disease.
premature coronary heart disease.
premature myocardial infarction in men.
Progressive AV-block and anomalous venous return
pronounced septal hypertrophy
proteinuria and cardiovascular disease
psychiatric manifestations of velo-cardio-facial syndrome
pulmonary arterial hypertension sclerosis, systemic
pulmonary fibrosis
pulmonary fibrosis sarcoidosis
pulmonary fibrosis; Pigeon breeders disease
pulmonary function
pulmonary function; nitric oxide; Pseudomonas aeruginosa infection
pulmonary hypertension
pulmonary hypertension; thrombosis, deep vein; pulmonary thromboembolism
pulmonary thromboembolism thromboembolism, venous
QT interval
radial artery hypertrophy
recurrent coronary event
reduced risk of restenosis after coronary stenting
respiratory-distress syndrome
restenosis
sclerosis, systemic
severe childhood thrombosis
SIDS/sudden infant death syndrome
sleep apnea
smoking
Spinal cord infarction and recurrent venous thrombosis
stroke
stroke, hemorrhagic stroke, ischemic
stroke, ischemic
stroke, ischemic; cerebrovascular disease; thrombosis, arterial
stroke, ischemic; fibrinogen
stroke, ischemic; stroke, hemorrhagic
stroke, lacunar
stroke, myocardial infarction
stroke; atherosclerosis
stroke; dementia
stroke; fibrinogen
subarachnoid hemorrhage
subclinical carotid atherosclerosis

	Takayasu arteritis
	Takayasu's arteritis
	thromboembolism
	thromboembolism, venous
	thromboembolism, venous; homocysteine; thromboembolism, arterial
	thromboembolism, venous; protein C
	thrombosis
	thrombosis, deep vein
	thrombosis, deep vein; Behcet's disease
	thrombosis, venous
	thrombosis; antiphospholipid syndrome
	throracic aortic aneurysm throracic aortic dissection
	transplant associated vasculopathy after cardiac transplantation
	Type II diabetes and hypertension
	unknown
	varicose ulcers
	vascular disease
	vascular endothelial growth factor
	vascular function; vascular morphology
	vascular NAD(P)H oxidase activity
	vasculitis, antineutrophil cytoplasmic; antibodies-associated small vessel
	vasoconstriction, coronary
	vasodilation during pregnancy
	vasodilation, flow-mediated
	vasopressor infusion requirement
	venous thromboembolism
	venous thrombosis
	ventricular arrhythmia, malignant
	X-linked dilated cardiomyopathy
**CHEMDEPENDENCY**	
	addiction to heroin
	Alagille syndrome
	alcohol abuse
	alcohol abuse cirrhosis, alcoholic pancreatitis, chronic
	alcohol abuse drug dependence
	alcohol abuse substance abuse
	alcohol abuse; blood pressure, arterial
	alcohol abuse; impulse control disorder
	alcohol abuse; neuroticism
	alcohol abuse; nicotine dependence
	alcohol abuse; smoking behavior
	alcohol craving
	alcohol dependence
	alcohol dependence drug dependence
	alcohol dependence; cocaine dependence
	alcohol dependence; drug dependence

alcohol dependency
alcohol effects
alcohol intake
alcohol use
alcohol withdrawal
alcohol withdrawal alcoholism
alcohol withdrawl seizure and delirium tremens
alcohol-dependence
alcoholic liver disease
alcoholism
alcoholism personality disorders
alcoholism; alcohol withdrawal
alcoholism; delirium tremens, alcohol-induced
alcoholism; pancreatitis; pancreatitis, alcoholic
alcoholism; renal disease, end stage
alcoholism-related traits
alcohol-related phenotypes nicotine
alcohol-withdrawal delirium
atriventricular block long QT syndrome
caffeine consumption
cirrhosis, alcoholic
cocaine dependence
cocaine dependence or abuse
cocaine dependence.
cocaine dependence/abuse
delirium tremens, alcohol-induced; alcohol withdrawal
dopamine release
drug abuse
heroin abuse
heroin addiction
heroin dependence
IgE
methamphetamine abuse
methamphetamine abuse psychoses
methamphetamine abuse schizophrenia
methamphetamine abuse; psychoses
methamphetamine abuse; substance abuse
methamphetamine dependence substance abuse
naltrexone response in alcohol-dependent patients
nicotine
nicotine dependence
nicotine dependence smoking behavior
nicotine smoking behavior
nicotine; personality trait
opiate dependence
paranoia
problem drug use

	severity of alcohol dependence
	smoking behavior
	smoking behavior; nicotine dependence
	smoking cessation
	smoking cues
	substance abuse
	substance dependence
**DEVELOPMENTAL**	
	abnormal urogenital development
	achondroplasia
	achromatopsia
	age of menarche
	agenesis of the corpus callosum
	Axial skeletal defects
	Beckwith Wiedemann syndrome
	bone size
	cleft lip
	cleft lip and palate
	cleft lip with cleft palate
	cleft lip with cleft palate cleft lip without cleft palate
	cleft lip with cleft palate cleft lip without cleft palate cleft palate
	cleft lip with cleft palate; cleft lip without cleft palate
	cleft lip with cleft palate; cleft lip without cleft palate; cleft palate
	cleft lip with or without cleft palate
	cleft lip with or without cleft palate; cleft palate, isolated
	club foot
	Coffin-Lowry syndrome.
	congenital adrenal hyperplasia
	congenital adrenal hyperplasia (CAH)
	congenital anomalies
	congenital anomalies; renal disease
	congenital bilateral absence of the vas deferens
	congenital central hypoventilation syndrome
	congenital contractural arachnodactyly
	cryptochidism
	cryptorchidism
	cryptorchidism.
	Down syndrome
	Familial spondyloepiphyseal dysplasia tarda, brachydactyly, and precocious osteoarthritis
	fetal growth
	fragile X syndrome
	fragile X-associated tremor/ataxia syndrome
	haptoglobin development
	height
	Hurler syndrome
	hypodontia

	hypospadias
	hypothyroidism; cleft palate, isolated; thyroid agenesis
	isolated penile hypospadias
	Left-right axis malformations
	limb deficiency anomalies
	Marfan phenotype
	Marfan syndrome
	mental disorder
	mental retardation
	micropenis
	multiple epiphyseal dysplasia
	muscular dystrophy
	neural tube defects
	neural tube defects and preeclampsia
	non-syndromic cleft lip and palate
	Noonan syndrome
	omphalocele
	Peutz-Jeghers syndrome
	Peutz-Jegher's syndrome
	postnatal growth
	precocious puberty
	salt-wasting congenital adrenal hyperplasia
	small for gestational age
	small-for-gestational-age
	Swyer syndrome
	talipes equinovarus
	WAGR syndrome
**HEMATOLOGICAL**	
	agranulocytosis
	albuminuria
	albuminuria; fibrinogen
	anemia, aplastic
	anemia, iron deficiency
	anemia, malaria related; malaria, cerebral
	anemia; thrombocytopenic purpura, idiopathic
	aplastic anaemia
	aplastic anemia, acquired
	Bernard-Soulier Syndrome
	bilirubin
	bleeding tendency
	blood and blood forming organ disorders
	C2-deficiency gene
	C4gene deletion
	chronic hemolytic anemia
	chronic non-spherocytic haemolytic anaemia.
	coagulation disorder

	coagulopathy
	factor V Leiden
	factor VII
	factor VII levels
	factor VIII and factor IX genes
	factor XII-deficient patients
	fibrinogen
	haptoglobin
	hematology indices
	hematopoietic progenitor cells, mobilization of
	hematopoietic stem cell transplantation
	hemophilia
	Increased platelet aggregability
	Langerhans cell histiocytosis
	malaria
	neonatal thrombocytopenia
	over anticoagulation
	P1 blood group
	placental vascular complications
	platelet aggregation; nitric oxide activity
	porphyria cutanea tarda
	sickle cell anemia
	thalassemia
	Thrombin activatable fibrinolysis inhibitor
	thrombin-activatable fibrinolysis inhibitor levels
	thrombocythemia
	thrombocytopenia
	thrombocytopenic purpura, iimmune
	thrombophilia
	Vitamin D deficiency rickets
**IMMUNE**	
	acute anterior uveitis
	acute graft-versus-host disease
	acute renal allograft rejection
	acute respiratory distress syndrome IL* production
	Addison's disease
	Addison's disease diabetes, type 1 Graves' disease rheumatoid arthritis
	Addison's disease Graves' disease
	Addison's disease Graves' disease lupus erythematosus polyangitis Wegener's granulomatosis
	Addison's disease; Graves' disease
	allele frequency/ normal
	Allergic asthma
	allergic bronchopulmonary aspergillosis ABPA
	allergic disease
	allergic diseases

	Allergic diseases (bronchial asthma. atopic dermatitis and/or food-related anaphylaxis)
	allergic rhinitis
	allergic rhinitis asthma
	allergic rhinitis dermatitis and eczema fatty acid
	allergies
	allergies; common cold
	allergy
	allergy asthma
	allergy dermatitis and eczema
	allergy, latex; latex allergy
	allograft dysfunction, renal
	allograft outcome
	alopecia areata
	alzheimer`s Disease
	ANCA positive patients
	ankylosing spondylitis
	ankylosing spondylitis independent of HLA-B27
	anti-cyclic citrullinated peptide antibodies rheumatoid arthritis
	anti-GAD65 antibody
	antineutrophil cytoplasmic antibody; (ANCA)-associated vasculitis
	antiphospholipid syndrome
	antiphospholipid syndrome thrombosis
	anti-Ro 52-kd autoantibodies
	arthritis
	arthritis lupus erythematosus
	arthritis lymphopenia nephritis, lupus
	arthritis, juvenile
	arthritis, psoriatic
	arthritis, rheumatoid
	arthritis; Familial Mediterranean Fever
	Aspirin-induced asthma
	asthma
	asthma (childhood)
	asthma and atopy
	asthma and IgE in childhood
	asthma asthma, aspirin-intolerant
	asthma atopy
	asthma atopy IgE
	asthma bronchodilator response IgE lung function
	asthma dermatitis and eczema
	asthma eczema
	asthma IgE
	asthma IgE levels
	asthma IgE wheezing
	Asthma in men
	asthma respiratory syncytial virus

asthma rheumatoid arthritis
Asthma severity
Asthma smokers
asthma, aspirin-induced
asthma, aspirin-intolerant
asthma, dust mite-sensitive
asthma.
Asthma. asthma severity
Asthma. atopy
Asthma. atopy. atopic dermatitis
Asthma. atopy. spIgE.
Asthma. BHR. Total IgE. SPT
asthma. FEV(1)
Asthma. FEV1
Asthma. rhinitis
asthma; allergic disease
asthma; allergic disease, IgE mediated
asthma; allergic rhinitis
asthma; allergic rhinitis; atopic dermatitis
asthma; allergies
asthma; allergy
asthma; aspirin intolerance
asthma; atopic dermatitis
asthma; atopy
asthma; atopy; dermatitis and eczema
asthma; chronic obstructive pulmonary disease/COPD
asthma; dermatitis and eczema
asthma; dermatitis and eczema; anaphylaxis, food-related
asthma; dermatitis and eczema; rhinitis
asthma; IgE
asthma; respiratory syncytial virus; juvenile arthritis
asthma; urticaria
atherosclerosis, coronary
Atopic asthma
atopic dermatitis
atopic dermatitis.
Atopic dermatitis. atopy
atopic eczema
Atopy
Atopy (IgE)
Atopy (total & specific IgE)
atopy IgE urticaria, aspirin-intolerant
atopy. asthma. Netherton
atopy; IgE levels
atopy-susceptibility
Autoimmune Hepatitis
Autoimmune hepatitis type 1

autoimmune hypothyroidism
autoimmune inflammatory disease
autoimmune myasthenia gravis
autoimmune pancreatitis
autoimmune polyglandular syndrome
autoimmune response
autoimmune thyroid disease
autoimmune thyroid disease; thyroid disease, autoimmune
autoimmune thyroiditis Graves' disease Hashimoto's thyroiditis
autologous mixed lymphocyte reaction
Behcet's disease
Behcet's disease thromboembolism, venous
Behcet's disease; vasculitis
beta-cell function; diabetes, type 1
betaCL osteocalcin
BHR
bone marrow transplantation
bronchial asthma
Bronchial asthma (childhood & adult)
Bronchial asthma (childhood only)
bronchial hyperresponsiveness
bronchopulmonary dysplasia respiratory distress syndrome, neonatal
bullous pemphigoid
CD14 expression
celiac
celiac disease
celiac disease lupus erythematosus rheumatoid arthritis
cerebral malaria
childhood atopic asthma
Chinese ankylosing spondylitis patients
cholangitis, sclerosing
cholangitis, sclerosing Crohn's disease inflammatory bowel disease
cholangitis, sclerosing Crohn's disease ulcerative colitis
cholesterol, LDL; cholesterol, total; C-reactive protein; apoA2; apoB
chronic bronchitis
chronic GVHD
chronic immune thrombocytopenic purpura.
chronic obstructive pulmonary disease
chronic obstructive pulmonary disease/COPD
chronic obstructive pulmonary disease/COPD; bronchiectatic disease
chronic obstructive pulmonary disease/COPD; emphysema
chronic pancreatitis
chronic pancreatitis.
chronic periodontitis
chronic periodontitis.
cirrhosis, biliary primary hepatitis, autoimmune
Coeliac

coeliac disease
collagen disease juvenile arthritis rheumatoid arthritis Still's disease
Contact Allergy
contact hypersensitivity
Contact Sensitisation
C-reactive protein
C-reactive protein (CRP) concentration
C-reactive protein glycation inflammation insulin oxidative stress prostaglandin tumor necrosis factor alpha
Crohn's disease
Crohn's disease and IBD
Crohn's disease and ulcerative colitis
Crohn's disease inflammatory bowel disease ulcerative colitis
Crohn's disease rheumatoid arthritis sclerosis, systemic
Crohn's disease ulcerative colitis
Crohn's disease ulcerative colitis,Crohn's disease
Crohn's disease; ulcerative colitis
Crohn's disease; ulcerative colitis; inflammatory bowel disease
Decreased airway responsiveness
dermatitis and eczema
dermatitis herpetiformis.
dermatitis, atopic
dermatomyositis/ polymyositis
diabetes, latent autoimmune
diabetes, type 1
diabetes, type 1 diabetes, type 2 diabetic nephropathy retinopathy, diabetic
diabetes, type 1 diabetic nephropathy
diabetes, type 1 juvenile arthritis
diabetes, type 1 rheumatoid arthritis
diabetes, type 1 thyroid autoimmunity
diabetes, type 1 Vitamin D
diabetes, type 1; Addison's disease
diabetes, type 1; Addison's disease; Graves' disease; thyroiditis, chronic lymphocytic
diabetes, type 1; blood group incompatibility
diabetes, type 1; carotid atherosclerosis
diabetes, type 1; celiac disease
diabetes, type 1; IL-1RI
diabetes, type 1; nephropathy in other diseases
diabetes, type 2
diabetes-associated autoantibodies
DRS
early onset of multiple sclerosis.
early onset periodontitis
early polyarthritis
eczema
emphysema

EO
eosinophilia
familial mediterranean fever
FEV1
Fuchs heterochromic cyclitis
glomerulonephritis, Hepatitis B virus-associated
graft occlusion, atherosclerotic
graft versus host disease
graft-versus-host disease
Grave`s disease
Graves disease
Graves' disease
Graves' disease Hashimoto's thryoiditis
Graves' disease Hashimoto's thyroiditis
Graves' disease IgE
Graves' disease myasthenia gravis
Graves' disease; Hashimoto's thyroiditis
Graves' disease; ophthalmopathy, Graves'
Graves' disease; thyroiditis, chronic lymphocytic
Graves' hyperthyroidism
GVHD
Haemophilia with Chronic synovitis
Hashimoto's thryoiditis
hematology indices
hepatitis type 2, autoimmune
hepatitis, autoimmune
high interleukin-1 beta plasma levels
HIV-1 infection
Host defense and inflammatory outcomes
H-Thyroiditis
hyper-IgE syndrome and severe eczema. atopy
Hyper-IgE syndrome. severe eczema. atopy
hyper-IgM syndrome can form oligomers and trigger CD40-mediated signals
Hyperresponsiveness
hypothyroidism, autoimmune
hypothyroidism, goitrous juvenile autoimmune
idiopathic chronic pancreatitis
IgA deficiency
IgA nephropathy
IgD
IgE
IgE levels
IgE response
IgE response IgG
IgE, cord blood
IgE. SPT
IGF-I

	IGF-I levels; IGFBP-3 levels
	IL-18 concentration physical functioning
	IL18 expression level
	IL-18 lupus erythematosus
	IL-4
	IL-4 production
	Increased expression of the G gamma and A gamma globin
	Increased IgE
	increased interleukin-10 (IL-10) plasma levels
	inflammation
	inflammatory bowel disease
	inflammatory bowel disease/UC
	inflammatory myopathies
	inflammatory response
	inflammatory response in coronary bypass
	inflammatory urogenital disease
	interferon response
	interleukin-1 beta (IL-1 beta) synthesis capacity
	irritable bowel syndrome
	juvenile arthritis
	juvenile idiopathic arthritis
	kawasaki disease
	kidney transplant
	kidney transplant complications
	latex allergy
	latex-fruit syndrome
	lep. Leprosy
	leukemia virus type I
	Lupus
	lupus erythematosus
	lupus erythematosus multiple sclerosis
	lupus erythematosus periodontitis
	lupus erythematosus; nephritis, lupus
	lupus erythematosus; nephropathy in other diseases
	lupus erythematosus; periodontitis
	lupus erythematosus; rheumatoid arthritis
	lupus erythematosus; rheumatoid arthritis; cholangitis, sclerosing; celiac disease; juvenile arthrit
	lupus erythematosus; rheumatoid arthritis; Sjogren's syndrome
	lupus erythematosus; rheumatoid arthritis; Sjogren's syndrome; tuberculosis
	lupus erythematosus; sclerosis, systemic
	Lupus Nephritis
	malaria, cerebral
	microscopic polyangiitis
	Mite-sensitive asthma
	multiple sclerosis
	multiple sclerosis rheumatoid arthritis

multiple sclerosis(PPmultiple sclerosis)
myasthenia gravis
myelopathy, HTLV-1 associated
nephritis, lupus; glomerular microthrombus
nephropathy in other diseases
nephropathy, diabetic; diabetes, type 1
nephropathy, IgA
neutrophil immunodeficiency syndrome
nitric oxide, exhaled
Nocturnal asthma
nonatopic asthma and food allergy
Normal volunteers
nut allergy
osteomyelitis
pancreatitis
pancreatitis, acute pancreatitis, chronic
pancreatitis, autoimmune
pancreatitis, autoimmune; pancreatitis, chronic calcifying
pancreatitis, chronic
pancreatitis, tropical calcific
pemphigus
pemphigus foliaceus
pemphigus vulgaris
periodontal disease
periodontitis
periodontitis, early-onset
Physician diagnosed asthma
pneumoconiosis
pollen allergy
pollen-induced allergic rhinitis
polymyositis; dermatomyosis
postoperative systemic inflammatory reaction
Primary Biliary Cirrhosis
Primary Billiary Cirrosis
Primary Sclerosing Cholangitis
psoriasis
psoriasis psoriatic arthritis
psoriasis; dermatitis and eczema
psoriatic arthritis
purpura, allergic
reactive arthritis
Reiter's syndrome
renal disease
retinopathy, diabetic; nephropathy in other diseases
rheumatic diseases
rheumatic fever
rheumatic heart disease

rheumatoid arthritis
rheumatoid arthritis thyroid disease, autoimmune
rheumatoid arthritis; celiac disease
rheumatoid arthritis; Crohn's disease; systemic lupus erythematosus
rheumatoid arthritis; juvenile arthritis
rhinitis
rickets
sarcoidosis
sarcoidosis uveitis
sarcoidosis; tuberculosis
scleroderma
sclerosis, systemic
Semple rabies vaccine-induced autoimmune encephalomyelitis
Sepsis
Septic Shock
serum IgE levels
severe ulcerative colitis
silicosis
Sjogrens syndrome
Sjogren's syndrome
Sjogren's syndrome; purpura
SLE
spondyloarthropathies
SPT
SPT (cockroach)
SPT (HDM)
staphylococcal infection
Steroid-requiring asthma in sedentary women
Stevens-Johnson syndrome
Still's disease
sulfasalazine, adverse effects of
susceptibility to autoimmune disease
systemic inflammatory hyporesponsiveness
systemic inflammatory response syndrome
systemic juvenile idiopathic arthritis
systemic lupus erythematosus
Systemic Lupus Erythematosus (SLE)
systemic lupus erythematosus; arthritis, rheumatoid
systemic lupus erythematosus; lupus nephritis
Systemic responsiveness to lipopolysaccharide
systemic scleroderma
systemic sclerosis
thimerosal sensitization
thrombosis, deep vein; Behcet's disease
thryoiditis, chronic lymphocytic
thyroid autoimmunity
thyroiditis, chronic lymphocytic

	thyroiditis, Hashimoto's
	tIgE
	Total IgE
	Total IgE. atopic asthma
	Total IgE. Eosinophilia. DRS
	Total IgE. SPT. FEV1
	Total serum IgE
	total serum IgE levels
	Total serum IgE. atopic dermatitis
	Total serum IgE. specific IgE. AR
	Tropical calcific pancreatitis
	Tumor necrosis factor receptor-associated periodic syndrome
	type 1 diabetes
	ulcerative colitis
	ulcerative colitis and Crohn's disease
	ulcerative colitis; cholangitis, sclerosing
	ulcerative colitis; inflammatory bowel disease
	vitiligo
	vitiligo; autoimmune disease
	Vogt-Koyanagi-Harada syndrome
	Vogt-Koyanagi-Harada's disease
	X-linked lymphoproliferative disease
**INFECTION**	
	acne
	AIDS
	alveolar echinococcosis
	appendicitis
	aspergillosis
	bacteremia
	bacterial infection
	bacterial vaginosis
	bronchitis; pneumonia
	brucellosis
	Candida
	cerebral malaria
	cervical cancer
	cervical dysplasia H. pylori infection
	Chagas disease
	Chlamydia
	Chlamydia pneumoniae infection
	chronic active hepatitis C infection.
	chronic hepatitis C
	cirrhosis hepatitis C, chronic
	clinical tuberculosis
	Clostridium difficile toxin-induced diarrhea
	C-reactive protein Streptococcus pneumoniae

cystic fibrosis; P. aeruginosa infection
cytomegalovirus
cytomegalovirus leukemia
dengue disease
dengue hemorrhagic fever
duodenal ulcer gastritis
echinococcosis
febrile seizures
fibrosis hepatitis C, chronic
gastric atrophy H. pylori infection
graft-versus-host disease
H. pylori infection
HCV infection
Helicobacter pylori cagA subtype infection
Helicobacter pylori infection
Helicobacter pylori infection; gastritis, Helicobacter pylori-induced
hepatitis
hepatitis A vaccination, humoral immune response
hepatitis B
hepatitis B, chronic
hepatitis B, intrauterine
hepatitis C
hepatitis C infection
hepatitis C virus infection
hepatitis C, chronic
hepatitis C; hepatitis B
hepatitis E
hepatitis, fulminant non-A, nonB
herpesvirus, Kaposi sarcoma-associated
HIV
HIV disease
HIV disease progression
HIV infection
HIV; cytomegalovirus retinitis
HIV; pneumocystis carinii pneumonia
HIV; sclerosis, systemic
HIV-1
HIV-1 disease
HTLV-1 associated myelopathy/tropical spastic; paraparesis
HTLV-1 infection
human papillomavirus infection
interstitial cystitis
kidney transplant
Legionnaire's disease
Leishmania chagasi infection
leishmaniasis, cutaneous
leprosy

leprosy tuberculosis
leprosy type
liver disease
lung function
malaria
Malaria infection
malaria, cerebral
malaria, plasmodium falciparum
malaria; hypoglycemia; hyperparasitemia
malaria; schistosomiasis
malarial anemia and cerebral malaria
measles antibody level
meningococcal disease
meningococcal disease.
microscopy-positive tuberculosis
mucocutaneous leishmaniasis
multiorgan failure septic shock mortality
neonatal infection
neonatal sepsis
Nonsurvivors of sepsis (male only)
ocular Chlamydia trachomatis infection
ovalocytosis and malaria susceptibility
pancreatic necrosis pancreatitis, acute
papillomavirus infection
Parasitic infection
parvovirus
parvovirus B19 infection
placental malaria
Plasmodium falciparum reinfections
pneumococcal empyema pneumonia
pneumoconiosis, coal workers'
pneumoconiosis, coal workers' silicosis
pneumonia
Postoperative infectious complications
posttreatment Th2 immune response to S. mansoni Ags
Progression to AIDS
pulmonary tuberculosis
pyelonephritis vesicoureteral reflux
respiratory syncytial virus
respiratory syncytial virus disease
respiratory syncytial virus infection
rickets
SARS (severe acute respiratory syndrome)
SARS infection
schistosomal worm antigen
sepsis
sepsis; lung injury, acute

	sepsis; pneumonia; acute respiratory distress syndrome
	sepsis; serum tumor necrosis factor-alpha levels
	sepsis; systemic inflammatory response syndrome
	Septic Shock
	severe acute respiratory syndrome
	severe malaria
	severe RSV bronchiolitis
	Severe sepsis
	Severe sepsis/nonsurvival
	susceptibility or resistance to immunodeficiency virus type 1 infection
	susceptibility to tuberculosis
	trypanosomiasis
	tuberculoid type of leprosy
	tuberculosis
	tuberculosis disease
	tuberculosis meningitis
	tuberculosis tumor necrosis factor-alpha
	typhoid fever
	urinary tract infection
	vaginal micro-flora
	viral clearance
**METABOLIC**	
	1-hydroxypyrene, urinary
	1-hyrdoxypyrene glucuronide concentrations
	2-Amino-1-methyl-6-phenylimidazo[4,5-b]pyridine
	abdominal fat fatty acid glucose tolerance insulin lipids prostaglandin
	acid lipase deficiency and cholesterol ester storage disease
	adipocyte size, large subcutaneous abdominal
	adiponectin
	adiponectin glucose insulin lipids
	adiponectin hypertension plasminogen activator inhibitor-1
	adiponectin insulin
	adipose tissue lipoprotein lipase activity and lipoprotein lipid and glucose concentrations
	Adiposity and Abdominal Obesity
	adrogen body mass insulin polycystic ovary syndrome
	adult height urinary pyridinoline excretion
	aerobic exercise capacity
	age-related skeletal disorders
	albumin bone density calcium fractures, vertebral hyperparathyroidism kidney stone disease
	albuminuria
	albuminuria among aboriginal Australians
	albuminuria hypertension
	aldosterone
	aldosterone angiotensin II hypertension
	aldosterone blood pressure, arterial kidney function left ventricular function

aldosterone hypertension
aldosterone responsiveness to angiotensin
aldosterone responsiveness to the renin-angiotensin system
alpha-2-macroglobulin concentration obesity
altered B vitamin/thiol metabolism
altered lipid levels
altered lipid oxidation and early insulin secretion
amitriptyline metabolism
amitriptyline; nortriptyline
amyloidosis
amyloidosis Familial Mediterranean Fever
amyloidosis; Familial Mediterranean Fever
androgen
androgen deprivation therapy response prostate specific antigen
androgen insensitivity syndrome
angiotensinogen concentrations
ANS function
anthropometric and metabolic traits, relative to cardiovascular risk
anthropometric parameters diabetes, type 2 insulin
anti-atherogenic lipoprotein profile
anticoagulant complications
anticoagulant complications; bleeding complications
antilipolytic insulin sensitivity
antioxidant status
antipsychotic agent-induced weight gain
ApoA5 diabetes, type 2 triglycerides
apolipoprotein A-I levels
atherosclerosis
BCHE serum levels
benzene toxicity
beta-cell function
beta-cell function; insulin
biliary atresia
bilirubin; hemolysis
bilirubin; irinotecan pharmacogenetics
birth weight
birth weight body mass
birth weight glucose
birth weight hypoglycemia
bleeding complications
blood pressure
BMI
body composition
body composition changes
body fat
body fat body mass diabetes, type 2 lipoprotein
body fat distribution

	body fat leptin
	body fat; insulin resistance
	body height
	body mass
	body mass bone density muscle testing
	body mass bulimia harm avoidance personality disorders
	body mass cholesterol cholesterol, HDL diabetes, type 2 leptin
	body mass cholesterol, HDL
	body mass cholesterol, HDL diabetes, type 2 glucose insulin metabolic syndrome triglycerides
	body mass cholesterol, HDL food consumption lipids
	body mass cholesterol, LDL glucose insulin
	body mass diabetes, type 2 insulin
	body mass diabetes, type 2 triglycerides
	body mass energy metabolism
	body mass fatty acid glucose tolerance lipoprotein
	body mass glucose tolerance glycemia insulin
	body mass glucose tolerance glycemia obesity, localized
	body mass height weight gain
	Body Mass Index
	body mass index and waist circumference
	body mass insulin
	body mass insulin waist circumference
	body mass leptin obesity
	body mass leptin obesity, localized
	body mass metabolic rate
	body mass metabolic syndrome
	body mass obesity
	body mass obesity triglycerides
	body mass paraoxonase activity polycystic ovary syndrome
	body mass; adiposity
	body mass; cholesterol, HDL; blood pressure
	body mass; cholesterol, HDL; insulin; folate; homocysteine
	body mass; cholesterol, HDL; triglycerides; diabetes, type 2; cholesterol, LDL; hypertension; atherosclerosis
	body mass; cholesterol, LDL; cholesterol, total; insulin; apoB
	body mass; cholesterol; cholesterol, HDL; triglycerides; blood pressure; insulin resistance
	body mass; cholesterol; diabetes, type 2; blood pressure
	body mass; cholesterol; triglycerides; blood pressure
	body mass; diabetes, type 2
	body mass; diabetes, type 2; energy expenditure
	body mass; diabetes, type 2; glucose tolerance; insulin
	body mass; diabetes, type 2; insulin; glucose; HbA(1c)
	body mass; glucose
	body mass; glucose tolerance; insulin; lipids; blood pressure, arterial; heart rate; autonomic nervous system

body mass; glucose tolerance; insulin; obesity
body mass; glucose; insulin secretion
body mass; hemoglobin A(1c)
body mass; hypertension; insulin sensitivity
body mass; insulin
body mass; insulin sensitivity
body mass; insulin; glucose
body mass; insulin; glucose; nervous system activity
body mass; insulin; lipids; obesity
body mass; insulin; lipids; obesity; glucose; blood pressure; cortisol, salivary; leptin; leptin; testosterone
body mass; leptin
body mass; leptin; fat mass
body mass; lipids
body mass; muscle testing; IGF-I
body mass; obesity
body mass; triglycerides; blood pressure, arterial
Body Weight
body weight height limb length
Body Weight Regulation
body weight; seasonal affective disorder
bone characteristics
bone density
bone density and spondylosis
bone density but not spondylosis
bone density dehydroepiandrosterone sulphate osteoporosis
bone density dihydrotestosterone estradiol sex hormone binding globulin testosterone
bone density fracture risk
bone density fracture risk osteoporosis, postmenopausal
bone density fractures, vertebral
bone density fractures, vertebral osteoporosis, postmenopausal
bone density height
bone density osteoarthritis osteoporosis
bone density osteocalcin
bone density; calcium; fractures, nonvertebral; milk intolerance
bone density; fractures
bone density; fractures, vertebral
bone density; height
bone density; height; PTH; pyridinoline, urinary
bone density; muscle strength
bone density; osteoporosis
bone density; spondylosis, lumbar
bone density; tricho-dento-osseous syndrome
bone fractures
bone loss
bone marrow transplant

bone mass
bone mass IGF-I levels sex steroid hormones
bone mass; osteoporotic fractures
bone metastases
bone mineral density
bone mineral density (BMD)
bone mineral density (BMD) / quantitative ultra sound (QUS)
Bone mineral mass
Bone Mineralization
calcium
Calcium Nephrolithiasis
carotid artery damage
catalase activity
central adiposity
choesterol absorption
cholangitis, sclerosing
cholelithiasis
cholelithiasis; bilirubin
cholestasis
cholestasis; biliary atresia
cholesterol
cholesterol cholesterol, HDL cholesterol, LDL
cholesterol cholesterol, HDL cholesterol, LDL cholesterol, VLDL lipoprotein triglycerides
cholesterol cholesterol, HDL cholesterol, LDL triglycerides
cholesterol cholesterol, LDL diabetes, type 2 insulin triglycerides
cholesterol cholesterol, LDL fibrinogen lipoprotein myocardial infarct nitrate triglycerides
cholesterol cholesterol, LDL lipids lipoprotein
cholesterol cholesterol, LDL triglycerides
cholesterol heart disease, ischemic
cholesterol metabolic syndrome triglycerides
cholesterol status
cholesterol, HDL
cholesterol, HDL cholesterol, LDL
cholesterol, HDL hypertriglyceridemia triglycerides
cholesterol, HDL obesity
cholesterol, HDL triglycerides
cholesterol, HDL; arterial-wall changes; cholesterol efflux
cholesterol, HDL; atherosclerosis, coronary
cholesterol, HDL; cholesterol, LDL
cholesterol, HDL; cholesterol, LDL; apoA1; apoB
cholesterol, HDL; cholesterol, LDL; cholesterol, total; apoA1; apoE; apoB-100
cholesterol, HDL; cholesterol, total; insulin; cortisol
cholesterol, HDL; cholesteryl ester transfer protein
cholesterol, HDL; C-reactive protein; carotid intima-media thickness
cholesterol, HDL; hypertension

cholesterol, HDL; lipids
cholesterol, HDL; lipoprotein, LDL
cholesterol, HDL; triglycerides
cholesterol, HDL; triglycerides; adiposity
cholesterol, HDL; triglycerides; cholesterol, LDL
cholesterol, HDL; triglycerides; cholesterol, LDL; cholesterol, total; glucose
cholesterol, HDL; triglycerides; cholesterol, LDL; lipoprotein; fatty acid; uric acid
cholesterol, HDL; triglycerides; coronary heart disease; cholesterol, LDL; cholesterol, total
cholesterol, HDL; triglycerides; lipids
cholesterol, HDL; triglycerides; personality trait
cholesterol, LDL
cholesterol, LDL sPLA2-IIA mass
cholesterol, LDL,cholesterol, LDL hypercholesterolemia
cholesterol, LDL; cholesterol, total
cholesterol, LDL; cholesterol, total; apolipoproteins
cholesterol, LDL; C-reactive protein; triacylglycerol
cholesterol, LDL; hypertension; C-reactive protein; fibrinogen; homocysteine; white blood cell count
cholesterol, LDL; insulin; metabolism disorders
cholesterol, LDL; lipoproteins
cholesterol, total
cholesterol; cholesterol, HDL; cholesterol, LDL; hypertension; lipoproteins; obesity
cholesterol; cholesterol, HDL; lipoprotein, LDL; triglycerides; hypertension
cholesterol; cholesterol, HDL; lipoprotein; lipids
cholesterol; cholesterol, LDL
cholesterol; cholesterol, LDL; campesterol; sitosterol
cholesterol; cholesterol, LDL; carotid atherosclerosis
cholesterol; cholesterol, LDL; lipoproteins; fatty acid
cholesterol; diabetes, type 2
cholesterol; diabetes, type 2; hypertension; nephropathy, diabetic; albuminuria
cholesterol; diabetes, type 2; insulin; glucose; insulin resistance; PC-1 protein content; systolic blood pressure
cholesterol; hypercholesterolemia; cholesterol, LDL; hypertriglyceridemia
cholesterol; insulin; obesity; blood pressure
cholesterol; lathosterol
cholesterol; triglycerides
cholesterol; triglycerides; diabetes, type 2; obesity
cholesterol; triglycerides; obesity
cholesteryl ester storage disease
cholesteryl ester transfer protein
chondrocalcinosis
chronic symptoms in pesticide-exposed workers
coke-oven toxicity
Colon Cancer

combined bone mass
combined pituitary hormone deficiency
coronary artery disease
cortisol
cortisol escape from dexamethasone and elevated glucose levels
cortisol response to opioid blockade
C-reactive protein cholesterol, HDL insulin
creatinine; protein excretion, urinary
CYP2A6 poor metabolizer phenotype
CYP2D6 poor metabolizer phenotype
cystinuria
decreased bone mass in patients
diabetes
diabetes mellitus
diabetes mellitus type I
Diabetes Mellitus. Obesity. and Lipaemia
diabetes or impaired glucose tolerance
diabetes, gestational
diabetes, gestational insulin
diabetes, neurological manifestations
diabetes, type 1
diabetes, type 1 diabetes, type 2
diabetes, type 2
diabetes, type 2 fatty acid triglycerides
diabetes, type 2 ghrelin insulin
diabetes, type 2 glucose insulin
diabetes, type 2 glucose insulin obesity
diabetes, type 2 glucose tolerance
diabetes, type 2 hypertension
diabetes, type 2 hypertension intima-media thickness mortality
diabetes, type 2 insulin
diabetes, type 2 insulin metabolic syndrome
diabetes, type 2 kidney failure, chronic
diabetes, type 2 metabolic syndrome
diabetes, type 2 metabolism syndrome
diabetes, type 2 obesity
diabetes, type 2 plasminogen activator inhibitor type 1 levels
diabetes, type 2 retinopathy, diabetic
diabetes, type 2 rheumatoid arthritis
diabetes, type 2 sleep apnea
diabetes, type 2; atherosclerosis, generalized
diabetes, type 2; body fat; lipid metabolism
diabetes, type 2; cardiovascular disease; birth weight
diabetes, type 2; cholesterol, LDL; glucose tolerance; blood pressure, arterial; glycosylated hemoglobin A1
diabetes, type 2; diabetes, type 1
diabetes, type 2; glucose tolerance

diabetes, type 2; glucose tolerance; obesity
diabetes, type 2; glucose; HbA1c
diabetes, type 2; hypertension
diabetes, type 2; hypertension; insulin
diabetes, type 2; hypertension; obesity, localized
diabetes, type 2; insulin
diabetes, type 2; insulin; glucose; C-peptide; fatty acid
diabetes, type 2; insulin; leptin
diabetes, type 2; insulin; lipids; obesity
diabetes, type 2; insulin; obesity
diabetes, type 2; metabolic syndrome
diabetes, type 2; myocardial infarction
diabetes, type 2; nephropathy
diabetes, type 2; nephropathy in other diseases
diabetes, type 2; nephropathy, diabetic
diabetes, type 2; obesity
diabetes, type 2; polymetabolic syndrome
diabetes, type 2; stroke, ischemic
diabetes, type 2; weight loss; weight gain
diabetic nephropathy; retinopathy, diabetic
dietary intake and body composition
drug genotoxicity
drug hypersensitivity
drug hypersensitivity; asthma; rhinosinuitis, allergic fungal; sinusitis
drug metabolism
dyslipidemia
dyslipidemia and insulin resistance
dyslipidemias
electrolyte levels
elevated ACE
elevated fasting blood glucose levels
elevated HDL cholesterol levels
endogenous hypertriglyceridemia and familial hypercholesterolemia
enzyme activity
exercise-mediated changes of insulin resistance
Fabry disease
familial combined hyperlipidaemia.
familial combined hyperlipidemia
familial defective apolipoprotein B
familial defective apolipoprotein B-100 in a Chinese man
familial defective apolipoprotein B100.
familial diabetes insipidus
familial hypercholesterolaemia
fasting total cholesterol and LDL-cholesterol concentrations only
fibrinogen
fibrinogen; Budd-Chiari syndrome
folate

	folate homocysteine
	folate homocysteine vitamin B12
	folate; homocystinuria
	G6PD deficiency
	gallstone disease
	gallstones
	Gaucher disease
	Gestational Diabetes
	gestational diabetes mellitus
	Gilbert syndrome
	Gilbert's syndrome
	glucocorticoid deficiency
	glucose
	glucose insulin
	glucose insulin obesity
	glucose intolerance
	glucose metabolism
	glucose response
	glucose tolerance
	glucose tolerance hyperglycemia
	glucose tolerance insulin
	glucose tolerance insulin lipemia lipid oxidation
	glucose tolerance triglycerides
	glucose tolerance; fatty acid
	glucose tolerance; insulin
	glucose tolerance; insulin; body fat
	glucose tolerance; insulin; obesity; energy expenditure
	glucose tolerance; insulin; polycystic ovary syndrome; androgen levels; anthropometric measuments
	glucose tolerance; insulin; vascular disease
	glucose tolerance; metabolic syndrome; adiponectin
	glucose; adiponectin
	glucose; insulin secretion
	glucose; paraoxonase-1
	glutamic acid decarboxylase antibodies
	glycogen storage disease
	gout
	growth hormone concentrations insulin-like growth factor
	growth hormone responsiveness
	growth hormone secretion and height
	HDL cholesterol/ BMI
	HDL-cholesterol
	HDL-cholesterol level
	Head and neck squamous cell cancer
	hemochromatosis
	Hemoglobin Stanleyville II
	hemolytic uremic syndrome

	high and low levels of cholesterol
	High Triglyceride/ Low HDL Cholesterol Levels
	high-density lipoprotein cholesterol level.
	higher body mass index
	higher levels of serum leptin
	hirsutism polycystic ovary syndrome
	homocysteine
	homocysteine nitric oxide
	homocysteine rheumatoid arthritis rheumatoid nodulosis
	homocysteine; vitamin B12; holotranscobalamin; methylmalonic acid
	homocystinuria
	homocyteine
	hormone disturbance
	hyperalphalipoproteinemia
	hyperandrogenism
	hyperbilirubinemia
	hypercalciuria
	hypercholesterolemia
	hypercholesterolemia of hypothyroidism
	hypercholesterolemia; cholesterol, LDL
	hypercholesterolemia; hyperlipidemia; hypertriglyceridemia; dysbetalipoproteinemia
	hyperglycemia insulin
	hyperhomocystinemia
	hyperinsulinemia; fat oxidation; intestinal fatty acid absorption
	hyperlipidemia
	hyperparathyroidism
	hypertension
	hypertriglyceridemia
	hypertriglyceridemia and the development of fatty liver
	hyperuricemia; uric acid
	hypoglycemia
	hypogonaotropic hypogonadism
	hypolactasia, adult-type
	hypopituitarism midline neurological abnormalities optic nerve hypoplasia pituitary dysfunction septooptic dysplasia
	hypospadias
	hypothyroidism
	hypothyroidism, congenital
	idiopathic haemochromatosis
	increased antilipolytic insulin sensitivity
	increased body weight and dyslipoproteinaemia involving triglyceride-rich lipoproteins
	increased concentration of HDL-C and decreased promoter activity
	increased fat oxidation and hyperinsulinemia
	increased fatty acid binding increased fat oxidation and insulin resistance
	Increased plasma concentrations of total cholesterol

	increased plasma leptin levels
	increased plasma triglyceride and lower high-density lipoprotein cholesterol concentrations
	increased plasma triglyceride levels
	increased prevalence and level of insulin autoantibodies
	increased sensitivity to glucocorticoids
	increased serum glucose concentrations
	increased serum iron transferrin saturation and hemoglobin
	Inflamatory Bowel disease
	inflammatory bowel disease; bone density
	insulin
	Insulin in Obese Women with Impaired Glucose Tolerance (IGT)
	insulin left ventricular mass
	insulin obesity
	insulin resistance
	insulin resistance and intra-abdominal fat thickness
	insulin resistance in obesity
	insulin resistance; fatty acid
	insulin response to oral glucose
	Insulin sensitivity
	insulin triglycerides
	insulin; glucose
	insulin; glucose; C-peptide; fatty acid
	insulin; lipids; glucose
	insulin; lipids; obesity; glucose; leptin
	insulin; lipids; obesity; glucose; leptin; cortisol
	insulin; lipoprotein; lipids
	insulin; obesity
	insulin; obesity; blood pressure, arterial; blood and blood forming organ disorders
	insulin; obesity; leptin
	insulin-like growth factor
	insulin-resistance syndrome
	Insulin-resistant diabetes
	intima-media thickness; carotid plaque
	iron levels
	iron levels; soluble transferrin receptor; transferrin
	iron load
	lactate dehydrogenase severe acute respiratory syndrome
	lactose intolerance
	LDL-cholesterol
	lead blood levels; zinc
	lead toxicity
	leanness obesity
	leisure physical activity
	leptin
	leptin expression

leptin levels
leptin; fat mass
Lesch-Nyhan syndrome
lipid levels
lipid metabolism
lipid metabolism disorders
lipid metabolism disorders; hyperlipidemia
lipid metabolism disorders; metabolic syndrome
lipid metabolism disorders; obesity
lipid profiles
lipids
lipids; atherosclerosis
lipids; C-reactive protein; obesity; glucose; leptin
lipids; height
lipids; obesity; glucose; cortisol
lipolysis
lipoprotein
lipoprotein level
lipoprotein levels
lipoprotein subclass profiles
lipoprotein(a) concentration
lipoprotein, LDL
lipoprotein; lipids
lipoproteins
liver disease; Wilson disease
low bone-mineral density and rapid postmenopausal bone loss
low insulin and cholesterol levels
Lp(a)
Lp(a) levels
mannose-binding lectin levels
mannose-binding lectin levels, serum
McArdle disease
medium-chain acyl-CoA dehydrogenase (MCAD) deficiency
medium-chain acyl-CoA dehydrogenase deficiency
metabolic syndrome
metabolic syndrome obesity
metabolism disorders
microalbuminuria
mild familial hypercholesterolaemia
minimum lifetime body mass index
myeloperoxidase activity
neonatal hyperbilirubinemia
nephrolithiasis
nephropathy
nephropathy in other diseases
nephropathy, diabetic

nephropathy, diabetic; retinopathy, diabetic; neuropathy, diabetic; microalbuminuria, diabetic
nitric oxide
normal serum cholesterol levels
obesity
Obesity ?????????
Obesity and insulin response
Obesity and Type 2 Diabetes
Obesity- associated hypertension ???????
obesity PAI-1 levels
obesity; affective disorder
obesity; birth weight
obesity; blood pressure, arterial
obesity; blood pressure, arterial; sleep apnea
obesity; cortisol
obesity; depression; cerebrovascular disease
obesity; energy expenditure
obesity; hyperandrogenism
obesity; metabolic syndrome
obesity; polycystic ovarian syndrome; hyperandrogenism
ossification of the posterior longitudinal ligament
ossification of the posterior longitudinal ligament of the spine
ossification of the posterior longitudinal ligament of the spine (OPLL)
osteoarthritis
osteonecrosis
osteopenia osteoporosis
osteoporosis
osteoporosis, postmenopausal
osteoporosis, postmenopausal; bone density
osteoporosis, postmenopausal; bone density; hormone disturbance
osteoporosis, postmenopausal; osteoporosis
osteoporosis; cirrhosis, primary biliary
oxidative stress
PAH metabolites, urinary
PAH-DNA adducts
PAI-1 levels
pesticide toxicity
phenylketonuria
plasma cholesterol levels and body mass index
plasma endothelin-1 levels
plasma factor VII activity and antigen levels
plasma fibrinogen levels
plasma fibrinogen levels in smokers and non-smokers
plasma glucose concentration
plasma HDL-cholesterol
plasma high density lipoprotein cholesterol and apolipoprotein AI
plasma high-density lipoprotein cholesterol levels

plasma homocysteine levels
plasma levels of homocysteine
plasma lipid and apolipoprotein levels
Plasma Lipid Levels
plasma lipid levels and body mass index
plasma lipoprotein(a) levels
plasma lipoproteins
plasma protein C levels
plasma protein C levels and thrombotic risk
plasma total and LDL-apolipoprotein B
plasma vWF
plasmatic PAI-1 activity
plasminogen activator; fibrinolytic activities
platelet alpha2 beta1 density
polycystic ovarian disease
polycystic ovarian syndrome
polycystic ovarian syndrome; insulin resistance
Polycystic ovaries and premature male pattern baldness
polycystic ovary syndrome
polycystic ovary syndrome.
polycystic ovary syndrome; adrenal androgen
polycystic ovary syndrome; diabetes
polycystic ovary syndrome; pregnancy loss, recurrent
Posttransplantation diabetes mellitus (PTDM)
primary biliary cirrhosis]
primary hyperparathyroidism
prolactin
reduced energy expenditure
reduced HDL cholesterol and LpA-I
retinopathy, diabetic
schizophrenia
serum androgen concentrations
serum cholesterol and LDL-cholesterol levels
serum concentrations of creatinine
Serum Insulin Levels in obese
serum lipid levels
serum low density lipoprotein
serum testosterone level
serum total and ionized calcium concentration
serum triglyceride levels
sex hormones
sex steroids
sex-dependent growth
sickle cell anemia
sickle cell disease
Sjogren-Larsson syndrome
skeletal responsiveness to estrogen

	soluble cytotoxic T lymphocyte-associated antigen-4
	sulfotransferases enzyme activity
	Tay-Sachs disease
	testosterone
	testosterone; prostate specific antigen
	thrombosis
	thyroid function
	total serum calcium
	triacylglycerol
	triglyceride levels
	triglycerides
	triglycerides; atherosclerosis, coronary
	triglycerides; cholesterol, LDL
	triglycerides; cholesterol, LDL; hyperlipidemia
	triglycerides; cholesterol, VLDL
	triglycerides; hypercholesterolemia
	triglycerides; insulin
	triglycerides; insulin; cardiovascular disease risk
	triglycerides; insulin; obesity
	triglycerides; obesity
	tuberculosis
	Type 2 Diabetes Mellitus
	Type 2 Diabetes (Retinopathy)
	Type 2 diabtes
	Type II diabetic
	type III hyperlipoproteinemia
	uric acid
	urinary calculus
	urinary PAH metabolites
	urolithiasis
	variation in plasma lipoproteins
	vasodilation
	very low bone mass
	waist circumference
	warfarin sensitivity
	warfarin therapy, response to
	weight gain
	weight gain (antipsychotic-drug induced)
	weight gain, antipsychotic drug-induced
	weight loss
	Wilson disease
**NEUROLOGICAL**	
	age-related macular degeneration
	ALS/amyotrophic lateral sclerosis
	Alzheimer`s Disease
	Alzheimers disease

	Alzheimer's Disease
	Alzheimer's disease cognitive function
	Alzheimer's disease dementia
	Alzheimer's disease dementia, vascular
	Alzheimer's disease depressive disorder, major
	Alzheimer's disease Parkinson's disease
	Alzheimer's Disease/Coronary artery disease
	Alzheimer's disease; Abeta load in brain
	Alzheimer's disease; aggressive behavior
	Alzheimer's disease; anxiety disorder; depression; psychoses; aggressive behavior
	Alzheimer's disease; atherosclerosis, coronary
	Alzheimer's disease; attention deficit disorder; conduct disorder; oppositional defiant disorder; dementia, frontotemporal
	Alzheimer's disease; cognitive function
	Alzheimer's disease; dementia
	Alzheimer's disease; dementia, vascular
	Alzheimer's disease; depressive disorder, major
	Alzheimer's disease; Parkinson's disease
	Alzheimer's disease; Parkinson's disease; multiple system atrophy
	Alzheimer's disease; Parkinson's disease; progressive supranuclear palsy; dementia, frontotemporal; multiple system atropy
	Alzheimer's disease; psychoses
	Alzheimer's disease; psychosis
	Alzheimer's disease; vascular endothelial growth factor levels
	argyrophilic grain disease
	ataxia (SCA)
	brain activity
	brain electrical response
	brain receptor-binding characteristics
	Charcot-Marie-Tooth disease
	Charcot-Marie-Tooth disease type 1A
	Charcot-Marie-Tooth disease type 1B
	Charcot-Marie-Tooth neuropathy type 1B
	Charcot-Marie-Tooth type 1 disease
	Charcot-Marie-Tooth type 1B
	chronic toxic encephalopathy
	clinically distinct Charcot-Marie-Tooth phenotype
	cluster headache
	Creutzfeldt-Jakob disease
	delayed sleep phase syndrome
	delayed sleep phase syndrome.
	dementia
	depression
	dyslexia
	dystonia
	dystonia, cervical

encephalopathies, transmissible spongiform
encephalopathy stroke
epilepsy
epilepsy, temporal lobe
epilepsy; seizures, febrile
epilpsy
essential tremor
essential tremor Parkinson's disease
event-related prefrontal activity
familial Alzheimer's disease associated
familial amyloid polyneuropathy.
familial amyloidotic polyneuropathy.
familial amyotrophic lateral sclerosis
familial amyotrophic lateral sclerosis.
Familial atypical progressive supranuclear palsy
frontotemporal dementia
frontotemporal dementia and Pick-like 3R and 4R tauopathy
Guillain-Barre syndrome
gyrate atrophy
hippocampal volume
Huntington disease
Huntington's disease
intelligence
Jakob-Creutzfeldt disease
Joubert syndrome
Joubert syndrome nephronophthisis
late-onset Alzheimer's disease.
Lewy Body Formation
memory disturbance
memory impairment
memory impairment, subjective
migraine
migraine migraine with aura
migraine risk
migraine with aura
motor neuron disease
Multiple sclerosis
narcolepsy
neuropathy
neuropathy, Alzheimer's disease related
neuropathy, diabetic
neuropathy, non-arteritic ischaemic optic
P300 amplitudes
pain response
panencephalitis, subacute sclerosing
Parkinsons disease
Parkinson's disease

	Parkinson's disease; alcohol abuse
	Parkinson's disease; cognition
	Parkinson's disease; dementia in other conditions
	Parkinson's disease; depression
	Parkinson's disease; hallucinations
	photoparoxysmal response
	prefontal cortex function
	prefrontal activity
	primary progressive aphasia
	progressive supranuclear palsy
	psychoses
	restless legs syndrome
	schizophrenia
	seizures
	seizures, febrile
	sleep disorders
	spastic paraplegia
	spinocerebellar ataxia
	spinocerebellar ataxia type 6
	tardive dyskinesia
	vertigo, migraine-associated
**PHARMACOGENOMIC**	
	11beta-hydroxylase activity
	18F-fluorodeoxyglucose uptake
	5-fluorouracil- related toxicity
	5-fluorouracil toxicity
	5-flurouracil toxicity
	ABT-773 plasma levels
	acenocoumarol pharmacokinetics
	acenocoumarol response
	acyl-mycophenolic acid-glucuronide
	adverse neonatal outcomes birth weight neuromotor symptoms respiratory distress syndrome, neonatal
	allergic reaction, betalactam
	alprazolam pharmacokinetics
	amlodipine pharmacokinetics
	amphetamine response
	analgesic response
	anemia C-reactive protein
	anesthesia effects diazepam pharmacokinetics
	anticoagulant complications; bleeding events, warfarin therapy-related; overanticoagulation, warfarin therapy-related
	anticoagulant response of acenocoumarol
	antiemetic response
	anxiety disorder
	arsenic metabolism
	arsenic toxicity

	asthma
	atrasentan phamacokinetics
	attention deficit hyperactivity disorder
	azathioprine adverse effects
	azathioprine tolerance
	benzene toxicity
	bilirubin
	bleeding complications
	blood pressure, arterial
	body mass
	body mass smoking behavior
	breast cancer
	breast cancer neutropenia
	carisoprodol metabolism
	carvedilol pharmacokinetics
	cervical cancer
	chemotherapy toxicity
	chlorpropamide pharmacokinetics
	cholestasis, drug-induced hepatotoxicity
	cholesterol
	cholesterol cholesterol, HDL cholesterol, LDL hyperlipidemia lipoprotein triglycerides
	cholesterol cholesterol, HDL cholesterol, LDL triglycerides
	cholesterol glucose insulin triglycerides
	cholesterol height
	cholesterol, HDL cholesterol, LDL heart transplant
	cholesterol, HDL triglycerides
	cholesterol, LDL
	citalopram adverse effects depressive disorder, major
	clomipramine metabolism
	colon cancer
	colorectal cancer
	coumarin sensitivity
	C-reactive protein cardiovascular event lipids
	Crohn's disease
	Crohn's disease ulcerative colitis
	cyclophosphamide bioactivation
	cyclophosphamide phamacokinetics
	cyclophosphamide pharmacokinetics
	cyclosporine nephrotoxicity
	cyclosporine pharmacokinetics
	cyclosporine resistance
	CYP2A6 phenotype
	depression
	depression sleep disorders
	depressive disorder, major
	diabetes, type 2

diabetes, type 2 insulin
digoxin, serum concentration
docetaxel pharmacokinetics
docetaxel pharmacokinetics docetaxel toxicity
dyskinesias, levodopa-induced
ebastine metabolism
edema
efavirenz pharmacokinetics HIV
efficacy and tolerability of simvastatin
epilepsy
epilepsy phentoin levels
esophagitis
extrapyramidal side effects
fentanyl effects
flubiprofen metabolism
fluvoxamine pharmacokinetics
gastrointestinal toxicity
glibenclamide pharmacokinetics lornoxicam pharmacokinetics
gliclazide pharmacokinetics
glycoprotein IIb/IIIa receptor activation platelet aggregation
growth response to growth hormone therapy
H. pylori infection
haloperidol, plasma
head and neck cancer
heart disease, ischemic hypercholesterolemia
hepatitis C, chronic
hepatotoxicity leukopenia
hepatotoxicity, diclofenac-induced
HIV
hypercholesterolemia
hypertension
ibuprofen clearance
indinavir pharmacokinetics
indisulam pharmacokinetics
irbesartan phamacokinetics
irinotecan pharmacokinetics
irinotecan pharmacokinetics irinotecan toxicity lung cancer
irinotecan pharmacokinetics lung cancer
irinotecan toxicity
irritable bowel syndrome
kidney cancer
kidney transplant; folate; homocysteine
lasoprazole pharmacokinetics
latanoprost efficacy
left ventricular ejection fraction
left ventricular fractional shortening
leukemia

leukemia, myeloid
lipids triglycerides
liver injury, drug-induced
liver transplant
loperamide concentration
loratadine pharmacokinetics
lornoxicam pharmacokinetics
losartan oxidation
lung cancer
methadone levels
methadone toxicity; etizolam pharmacokinetics
methotrexate efficacy
methotrexate toxicity
multiple chemical sensitivity
multiple sclerosis
mycophenolic acid pharmacokinetics
myocardial infarct
nateglinide pharmacokinetics
neurobehavioral response
nevaprine pharmacokinetics
nicotine
nicotine smoking behavior
obsessive compulsive disorder
omeprazole metabolism; sulfone metabolism
omeprazole pharmacokinetics
organophosphate toxicity
osteoporosis, postmenopausal
ovarian cancer
ovarian toxicity
ovarian toxicity, cyclophosphamide-related premature menopause, cyclophosphamide-related
paclitaxel pharmacokinetics
paclitaxel toxicity
pain intenity
pain response
phenobarbital pharmacokinetics phenytoin pharmacokinetics
phenprocoumon requirements
phenytoin
phenytoin levels
phenytoin metabolism
Plasma caffeine metabolite ratio
pravastatin kinetics
pravastatin pharmcokinetics
psychosis, methamphetamine
QT intervals
rabeprazole pharmacokinetics
rectal cancer

	rheumatoid arthritis
	rosiglitazone pharmacokinetics
	rosuvastatin pharmacokinetics
	saquinavir pharmacokinetics
	schizophrenia
	schizophrenia weight gain
	selenium
	simvastatin pharmacokinetics
	simvastatin treatment, efficacy and tolerability
	sirolimus pharmacokinetics
	smoking behavior
	suicide
	sulfonylurea failure, secondary
	tacrolimus pharmacokinetics
	tacrolimus pharmcokinetics
	topiramate pharmacokinetics valproate pharmcokinetics
	tuberculosis
	ulcerative colitis
	verapamil pharmacokinetics
	warfarin sensitivity
	warfarin therapy
	warfarin therapy, response to
	weight gain
	weight gain, antipsychotic-induced
	weight loss
**PSYCH**	
	ADHD
	ADHD and conduct disorder
	affective disorder
	affective psychoses
	Age-associated memory impairment
	aggression
	aggression, impulsivity, and central nervous system serotonergic responsivity
	aggressive behavior
	aggressive behavior of chondrosarcoma
	aggressive personality traits
	alcohol abuse; behavior problems
	anger-related traits
	anorexia nervosa
	anorexia nervosa and food intake
	anorexia nervosa bulimia
	anorexia nervosa obesity
	anorexia nervosa; bulimia
	anorexia nervosa; bulimia nervosa
	antidepressant medication intolerance.
	antisocial behavior conduct disorder

antisocial personality disorder
anxiety
Anxiety and hostility and depression
anxiety depression neuroticism psychological distress
anxiety disorder
anxiety disorder migraine
anxiety disorder panic disorder
anxiety disorder; alcohol abuse
anxiety disorder; blood pressure, arterial; memory impairment
anxiety disorder; depression
anxiety disorder; depressive disorder, major; neuroticism
anxiety disorder; depressive disorder, major; panic disorder
anxiety disorders
anxiety symptoms
Anxiety traits
anxiety-related temperament and behavior problems
anxiety-related traits
Asperger syndrome
attachment disorganization
attention deficit disorder; seasonal affective disorder
attention deficit hyperactivity disorder
attention deficit hyperactivity disorder behavior disorder
attention deficit hyperactivity disorder conduct disorder novelty seeking substance experimentation
attention deficit hyperactivity disorder depressive disorder, major
attention deficit hyperactivity disorder externalizing behavior IQ
attention deficit hyperactivity disorder-like phenotype
attention problems
attentional network function
Attention-deficit/hyperactivity disorder
aura anxiety and depression
autism
autism obsessive compulsive disorder
autism spectrum disorder
behavior problems
behavioural traits
binge eating
bipolar affective disorder
bipolar affective disorder.
bipolar affective disorder; unipolar affective disorder
bipolar and unipolar disorder
bipolar disorder
bipolar disorder catatonia schizophrenia
bipolar disorder depression
bipolar disorder depressive disorder, major
bipolar disorder mood disorder schizophrenia
bipolar disorder schizoaffective disorder

bipolar disorder schizophrenia
bipolar disorder suicide
bipolar disorder unipolar disorder
bipolar disorder; affective psychoses
bipolar disorder; mania, antidepressant-induced
bipolar disorder; unipolar disorder
bulimia
bulimia eating disorder
bulimia harm avoidance personality traits
caffeine-induced anxiety.
childhood-onset mood disorders
clozapine response
cognitive function
cognitive function dementia
cognitive function schizophrenia
cognitive function schizotypy
cognitive impairment
conduct disorder
conduct disorder; oppositional defiant disorder; antisocial personality disorder
craving
decision making
decision-making memory impairment
delusional disorder
delusions hallucinations psychoses
dementia
dementia, frontotemporal
dementia, vascular
depressed suicide
depression
depression and response to antidepressant treatment
depression depressive disorder, major
depression sleep disorders
depression stroke
depression, interferon-induced
depression, psychosis and antipsychotic response
depression; thrombocyte MAO activity
depressive disorder, major
depressive disorder, major hippocampal volume
depressive disorder, major suicide
depressive disorder, major; alcoholism
depressive disorder, major; bipolar disorder
depressive disorder, major; phobia
depressive disorder, major; suicide
depressive episode, major
disorganized attachment behavior
disruptive behavior
eating disorder

eating disorders
eating disorders mood disorders schizophrenia substance abuse
externalizing behavior problems and associated temperament traits
externalizing behavior problems; hyperactivity
eye movement disturbances
hallucinations schizophrenia
harm avoidance
hypotension
impulse behavior
impulse control disorder
impulsive behavior
impulsive inpatients
impulsivity
impulsivity; response inhibition
intelligence
interpersonal sensitivity; paranoid ideation; psychoticism
irritability hostility impulsivity and memory
major and bipolar depressives
major depression
major depressive disorder
major psychoses
manic-depressive illness
memory
memory performance
migrane. typical
mood disorder
mood disorder.
mood disorders
mood response
neuroticism
neuroticism; agreeableness
novelty seeking
obsessive compulsive disorder
obsessive compulsive disorder Tourette syndrome
obsessive-compulsive disorder
panic disorder
pathologic gambling
pathological gambling
perceived parenting
performance on the Wisconsin Card Sorting Test
personality disorders
personality trait of spritual acceptance
personality traits
personality traits; illegal drug use
posttraumatic stress disorder
post-traumatic stress disorder
psychiatric diseases

psychoses
psychoses schizophrenia
psychoses; methamphetamine dependence
psychoses; neuroticism
psychosis
psychosis schizophrenia
psychosis, alcoholic
psychotic disorders
reasoning skills
response to clozapine
schizoaffective disorder; alcoholism; bipolar disorder; suicide
schizophrenia
schizophrenia substance abuse
schizophrenia tardive dyskinesia
schizophrenia; alcohol abuse; depressive disorder, major; dermal erythema; schizoaffective disorder
schizophrenia; alcoholism; bipolar disorder
schizophrenia; bipolar disorder
schizophrenia; bipolar disorder; psychoses
schizophrenia; body fat; weight gain
schizophrenia; depressive disorder, major; bipolar disorder; delusional disorder; psychosis
schizophrenia; panic disorder
schizophrenia; psychoses
schizophrenia; schizoaffective disorder
schizophrenia; schizoaffective disorder; affective disorder
schizophrenia; schizoaffective disorder; bipolar disorder
schizophrenia; schizoaffective disorder; weight gain
schizophrenia; tardive dyskinesia
schizophrenia; therapeutic response
schizotypal traits
seasonal affective disorder
self-harm behavior
sleep disorders; depressive disorder, major
somatic anxiety
stress
suicidal behavior
suicidal behavior in depressed patients.
suicide
suicide family history
suicide, alcohol-dependent
suicide; depression
suicide; personality disorders
tardive dyskinesia
temperament
tic disorder, chronic
Tourette syndrome

	vascular dementia
	violent suicidal behavior
	violent suicide
**RENAL**	
	acute and chronic kidney transplant outcome
	altered urinary albumin/creatinine values
	chronic renal failure
	chronic renal failure.
	diabetes, type 1 diabetic nephropathy
	diabetes, type 2
	diabetic nephropathy
	diabetic nephropathy and retinopathy.
	diabetic nephropathy diabetic neuropathy
	diabetic nephropathy.
	diabetic neuropathy
	diabetic polyneuropathy
	diabetic retinopathy
	Diabetic Retinopathy in Type 2 Diabetes
	diabetic retinopathy.
	end stage renal disease
	End- Stage Renal Disease (ESRD)
	Familial distal renal tubular acidosis
	glomerulonephritis
	glomerulopathy lupus nephritis
	glomerulosclerosis, focal
	Henoch-Schonlein purpura; nephritis, Henoch-Schonlein
	Human Renal Transplantation
	IgA nephropathy
	immunoglobulin A nephropathy
	kidney disease
	kidney disease; vascular disease
	kidney dysfunction
	kidney failure
	kidney failure, acute
	kidney failure, chronic
	kidney failure, chronic kidney transplant
	kidney size, newborn
	kidney stone disease
	kidney transplant
	kidney transplant rejection
	medullary sponge kidney disease
	minimal change nephrotic syndrome
	nephritis
	nephrogenic diabetes insipidus
	nephropathy
	nephropathy in diabetes mellitus

	nephropathy in other diseases
	nephropathy in other diseases; cerebrovascular disease
	nephropathy in other diseases; peripheral vascular disease
	nephropathy, basement membrane
	nephropathy, diabetic
	nephropathy, diabetic; proteinuria
	nephropathy, IgA
	nephrosis
	nephrotic syndrome
	peritoneal transport
	polycystic kidney disease
	polycystic kidney disease 1
	progressive renal damage
	purpura, Henoch-Schonlein
	renal disease
	renal disease, end stage
	renal disease, end stage vascular endothelial growth factor
	renal scarring urinary tract infection
	renal transplant
	Renal Transplant Survival
	renin activity salt sensitivity
	retinopathy, diabetic
**REPRODUCTION**	
	abruptio placentae
	adenomyosis endometriosis
	amenorrhea premature ovarian failure
	anovulation and fecundity
	azoospermia
	azoospermia infertility, male oligospermia
	azoospermia oligospermia
	azoospermia; oligospermia
	azoospermia; oligospermia; infertility, male
	birth weight
	birth weight fetal growth
	birth weight perinatal complications
	birth weight preterm delivery
	birth weight; cord blood IGF-II levels
	birth weight; placental weight
	birth weight; preterm delivery
	chorioamnionitis
	endometriosis
	endometriosis adenomyosis and leiomyomata.
	endometriosis.
	endometriosis; adenomyosis
	erectile dysfunction
	estradiol

	fetal loss, early
	gestational age
	grow intrauterine growth
	GSTM1 methylation infertility, male
	hyperglycemia hypertension, pregnancy induced preterm delivery
	idiopathic recurrent miscarriage.
	in vitro fertilization
	increased ovulation rate
	infant birthweight
	infant disorganization
	infertility, female
	infertility, male
	infertility, tubal factor
	intrahepatic cholestatis of pregnancy
	intrauterine growth
	invitro fertilization
	male infertility
	menarche
	menarche; menopause
	menopause
	menstrual cycle
	menstrual disorders
	ovarian hyperstimulation syndrome
	ovulatory dysfunctions
	placental abruption
	placental malperfusion pregnancy complications
	preeclampsia
	pre-eclampsia
	preeclampsia.
	preeclampsia; abruptio placentae
	preeclampsia; eclampsia
	preeclampsia; hypertension, pregnancy induced
	pregnancy loss
	pregnancy loss, recurrent
	pregnancy loss, recurrent; coagulation disorder
	pregnancy loss, recurrent; fetal loss
	pregnancy loss, recurrent; in vitro fertilization success
	premature ovarian failure
	premature ovarian failure primary amenorrhea
	premature ovarian failure; menopause, early
	premature pubarche
	premature rupture of membranes
	preterm birth
	preterm delivery
	preterm delivery small-for-gestational age
	shortened gestation
	spontaneous preterm birth

	spontaneous preterm delivery.
**VISION**	
	Adult vitelliform macular dystrophy
	advanced retinopathy of prematurity
	albuminuria retinopathy, diabetic
	amyloidotic vitreous opacities Asparagine for isoleucine at position 84
	atopic dermatitis, ocular
	Autosomal-dominant retinitis pigmentosa
	cataract glaucoma retinal detachment visual acuity Vogt-Koyanagi-Harada syndrome
	cataract, presenile
	choroidal neovascularization geographic atrophy macular degeneration
	cone degeneration
	cone-rod dystrophy
	cone-rod dystrophy macular dystrophy retinitis pigmentosa
	congenital cataracts
	Defective colour vision
	diabetes, type 2; retinopathy, diabetic
	glaucoma
	glaucoma, normal tension
	glaucoma, primary congenital
	glaucoma, primary open-angle
	glaucoma, primary open-angle ocular hypertension
	glaucoma, primary open-angle; glaucoma, normal tension
	glaucoma; glaucoma, primary open-angle
	Graves' disease ophthalmology
	Graves ophthalmology Graves' disease
	intraocular pressure
	macular and peripheral retinal degeneration
	macular corneal dystrophy
	macular corneal dystrophy.
	macular degeneration
	macular degeneration, age-related
	myopia
	normal tension glaucoma
	ocular albinism
	ocular cicatricial pemphigoid
	optic atrophy
	optic disc atrophy
	pathological myopia
	pattern dystrophy of the retina
	Peters' anomaly
	primary open-angle glaucoma
	proliferative vitreoretinopathy rhegmatogenous retinal detachment
	retinal artery occlusion
	retinal hemangioblastomas
	retinal vascular occlusion

retinitis pigmentosa
retinoblastoma
retinopathy of prematurity
retinopathy, diabetic
retinoschisis, x-linked
Usher syndrome
X linked juvenile retinoschisis
young-onset keratoconus
zonular pulverulent cataract
